# Syringic Acid in Health and Disease: Mechanisms, Biological Activities, and Future Perspectives

**DOI:** 10.3390/ijms27188385

**Published:** 2026-09-20

**Authors:** Saleh A. Almatroodi, Arshad Husain Rahmani

**Affiliations:** Department of Medical Laboratories, College of Applied Medical Sciences, Qassim University, Buraydah 51452, Saudi Arabia

**Keywords:** syringic acid, oxidative stress, inflammation, neuroprotection, cardioprotection, hepatoprotection, antidiabetic activity, nanoformulations

## Abstract

Regular consumption of dietary flavonoids and phenolic compounds has been associated with a reduced risk of disease development and progression. In this regard, syringic acid, a hydroxybenzoic acid, is present in fruits and vegetables and plays a vital role in disease prevention. It exhibits therapeutic potential for various chronic diseases through diverse molecular mechanisms, including modulation of cellular and molecular pathways, regulation of inflammation and oxidative stress responses, and maintenance of cellular and tissue architecture. In addition, syringic acid has been shown to modulate gene expression and enzyme activities associated with various chronic diseases. Moreover, preclinical studies, including both in vitro and in vivo investigations, have evidenced that it has significant hepatoprotective, antidiabetic, neuroprotective, cardioprotective, and other beneficial effects. Furthermore, its role in synergistic studies aimed at increasing the efficacy of other compounds/drugs has also been reported. Advances in nanotechnology-based formulations have also improved their stability, bioavailability, and therapeutic efficacy, thereby expanding their potential biomedical applications. This review aimed to compile information on its sources, role in different pathogeneses, nanoformulations, and synergistic effects with other drugs. Despite its significant role in preclinical studies, further research is needed to optimize dosage regimens and mechanisms of action, develop novel delivery systems, and conduct comprehensive clinical studies to translate its potential into disease management.

## 1. Introduction

Natural compounds and their bioactive constituents have been widely recognized for their substantial role in the prevention and management of various pathogenesis [1]. These compounds contain diverse classes of bioactive molecules, and their roles in disease prevention and treatment have been demonstrated through in vitro, in vivo, and clinical studies. Their mechanisms of action involve the modulation as well as regulation of varied biological processes and cellular signaling pathways [2]. Moreover, it has also been reported that consumption of natural compounds and their bioactive constituents can reduce disease development and progression.

Phenolic acids are organic compounds commonly distributed in nature and found in diverse sources, such as fruits, teas, vegetables, wine, and even their by-products [3]. Phenolic acids constitute around one-third of all phenolic compounds and are broadly classified as hydroxybenzoic acids and hydroxycinnamic acids. Hydroxybenzoic acids are derivatives of benzoic acid and possess a C6–C1 carbon skeleton containing seven carbon atoms. The principal members of this group include gallic acid, *p*-hydroxybenzoic acid, ellagic acid, salicylic acid, syringic acid, gentisic acid, protocatechuic acid, and vanillic acid. These compounds vary from one another based on the modification of the aromatic ring [4]. Syringic acid (SA) plays an important role in disease prevention and therapeutic intervention by regulating a range of physicochemical and biochemical processes, including oxidative stress, inflammatory pathways, and cellular signaling mechanisms. These actions contribute to the prevention and decrease in various pathological conditions. The protective role of syringic acid (SA) against L-arginine-induced acute pancreatitis (AP) has been assessed by biochemical and histopathological analyses. Compared to healthy controls, rats with AP showed a meaningfully high total oxidant status, oxidative stress index, and lipid hydroperoxides, along with clear decreases in total antioxidant status (TAS) and sulfhydryl group levels. SA treatment meaningfully lessened oxidative stress by reducing oxidative markers and increasing antioxidant defenses. Overall, these results indicate that SA enhances antioxidant capacity and employs a protective effect against L-arginine-induced acute pancreatitis in rats [5]. SA exhibits a wide range of biomedical effects, including antioxidant, anti-inflammatory, and anticancer [6,7,8]. The study inspected the hepatoprotective role of SA against doxorubicin (DOX)-caused hepatic injury in rats. Histopathological, molecular, as well as biochemical assessments were conducted one month following the SA final administration. The results confirmed that SA meaningfully alleviated DOX-caused hepatotoxicity by restoring biochemical parameters, reducing oxidative stress, and suppressing inflammatory responses. These findings advocate that SA exerts a protective role against DOX-induced liver damage [9].

## 2. Methodology

A wide-ranging literature search was performed to identify published studies examining the mechanisms, therapeutic activities, sources, synergistic effects, safety, toxicity and nanoformulations of syringic acid. The search was performed using chief electronic databases, PubMed, Scopus, Web of Science, and Google Scholar, covering the period from July 1994 to June 2026. The main search keywords included syringic acid, antioxidant, anti-inflammatory, hepatoprotective, antidiabetic, neuroprotective, cardioprotective, anticancer, antimicrobial activity, mechanism of action, synergistic effects, nanoformulation, pharmokinetics and bioavailability. Both in vitro as well as in vivo experimental studies, as well as relevant human clinical studies, were included. Research articles and appropriate review articles were included in this article. Excluded studies were duplicated publications, non-English-language articles, conference abstracts, editorials, and case reports. Initially, 145 publications were recognized by the database search. After removal of duplicate publications, 134 publications were considered for evaluation and were included in the current review based on its objectives.

## 3. Structure, Sources, and Biosynthesis of Syringic Acid

**Figure 1:**
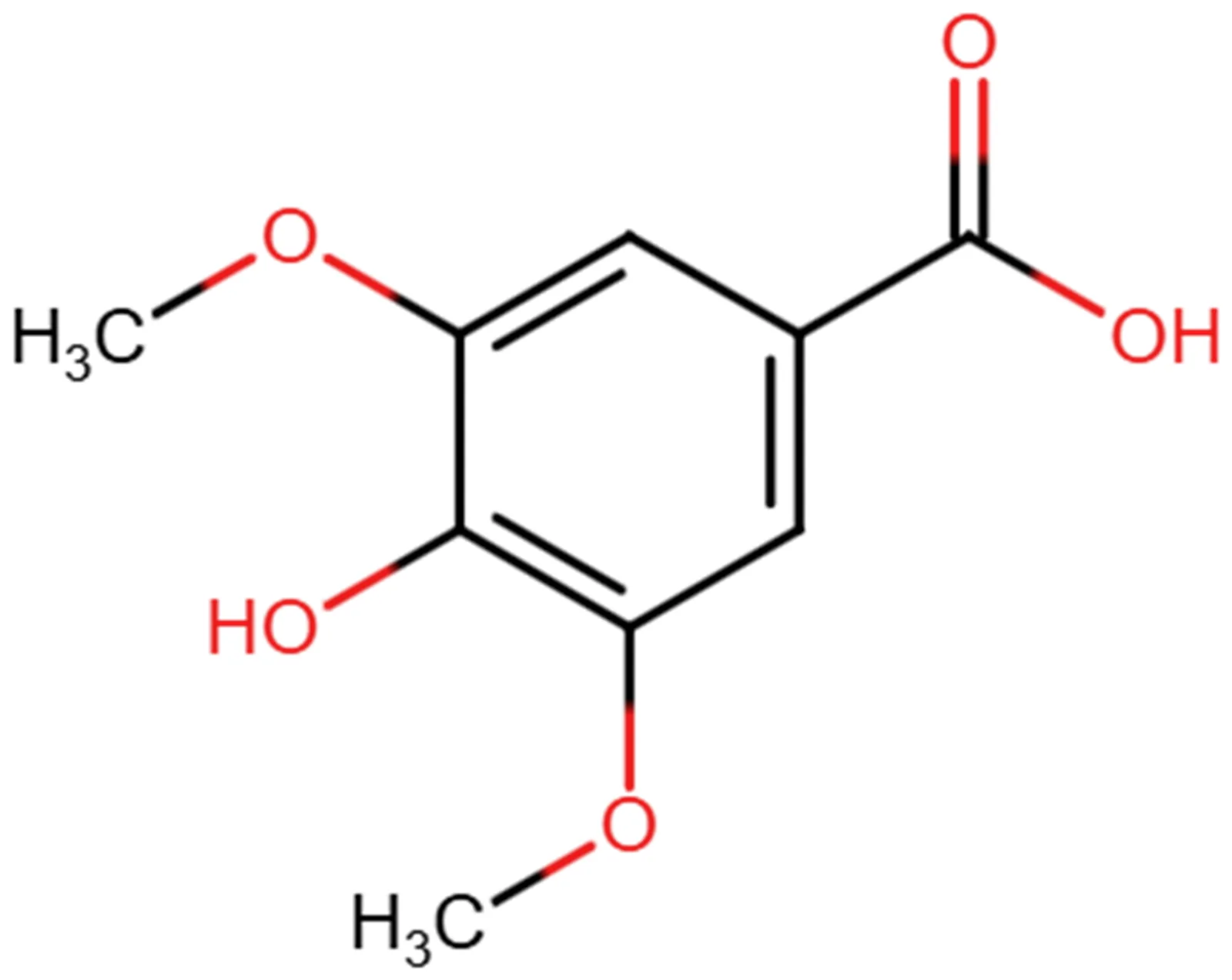
Chemical structure of syringic acid.

**Figure 2:**
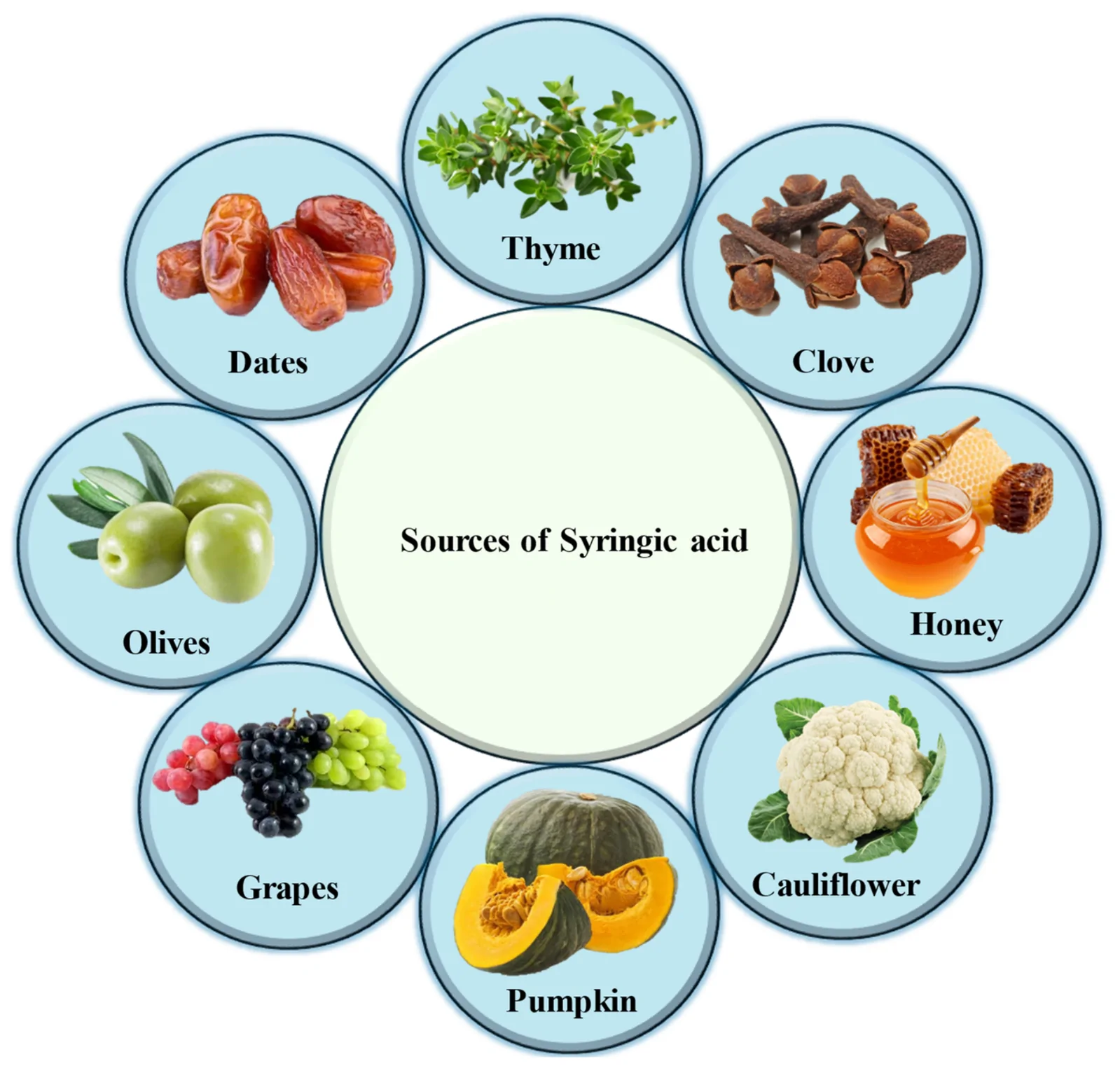
Dietary sources of syringic acid. The figure illustrates common foods and spices reported to contain syringic acid, including thyme, clove, honey, cauliflower, pumpkin, grapes, olives, and dates, with the chemical structure of syringic acid shown at the center.

SA is a naturally occurring phenolic acid [Figure 1] and a 3,5-dimethoxyphenolic derivative of gallic acid. The structural composition of SA is one benzene ring comprising two methoxy (-OCH3) groups, one hydroxyl (-OH) group, and one carboxyl (-COOH) group [10]. Syringic acid is found in many plant sources [Figure 2]. Various vegetables and fruits are rich sources of syringic acid, including dates, olives, grapes, pumpkins, cauliflower, spices, acai palm, honey, red wine, and vinegar [11,12,13,14]. Other sources of syringic acid are Raphanus sativus, Hemidesmus indicus, and Tagetes erecta flowers [15,16,17]. It also occurs in Chinese catalpa, garden balsam, New Jersey tea, thyme, rosemary [18], pot marigold [19], and clove [20].

Syringic acid (SA) is a vital phenolic acid that has attracted substantial attention because of its biological properties. In plants, SA is synthesized by a series of enzymatic reactions via the shikimic acid pathway [21], which acts as a substrate in protein synthesis and the formation of phenolic compounds [22]. The biosynthesis of SA involves several enzymatic steps beginning with phenylalanine and finally involving the methylation of sinapic acid, leading to the formation of SA [23]. The biosynthesis of SA is therefore enzymatically regulated and considerably dependent on plant species and environmental conditions. Its relatively simple and well-defined chemical structure allows chemical synthesis. However, pharmaceutical-scale production requires the optimization of yield, purity, and stability, meaning that simple chemical synthesis does not necessarily ensure straightforward medical application. Biotechnological production, together with formulation as well as nanodelivery strategies, may provide hopeful approaches for bioavailability improvement and therapeutic translation of SA.

## 4. Health Benefits and Disease Management Potential of Syringic Acid

Syringic acid (SA), a naturally occurring phenolic compound, has arisen as a promising bioactive molecule with a wide range of pharmacological effects. Growing experimental evidence highlights its potential in disease prevention and treatment, mainly through its ability to counteract oxidative stress and modulate inflammatory signaling pathways. These multifaceted biological activities have positioned SA as a promising candidate for developing therapeutic approaches targeting various oxidative stress- and inflammation-related disorders. Studies have also described its protective role in maintaining organ and tissue architecture. Growing evidence supports the beneficial effects of SA in the management of various chronic diseases. This review summarizes and describes the roles of SA in different pathogeneses below.

### 4.1. Anti-Inflammatory Potential of SA

**Figure 3:**
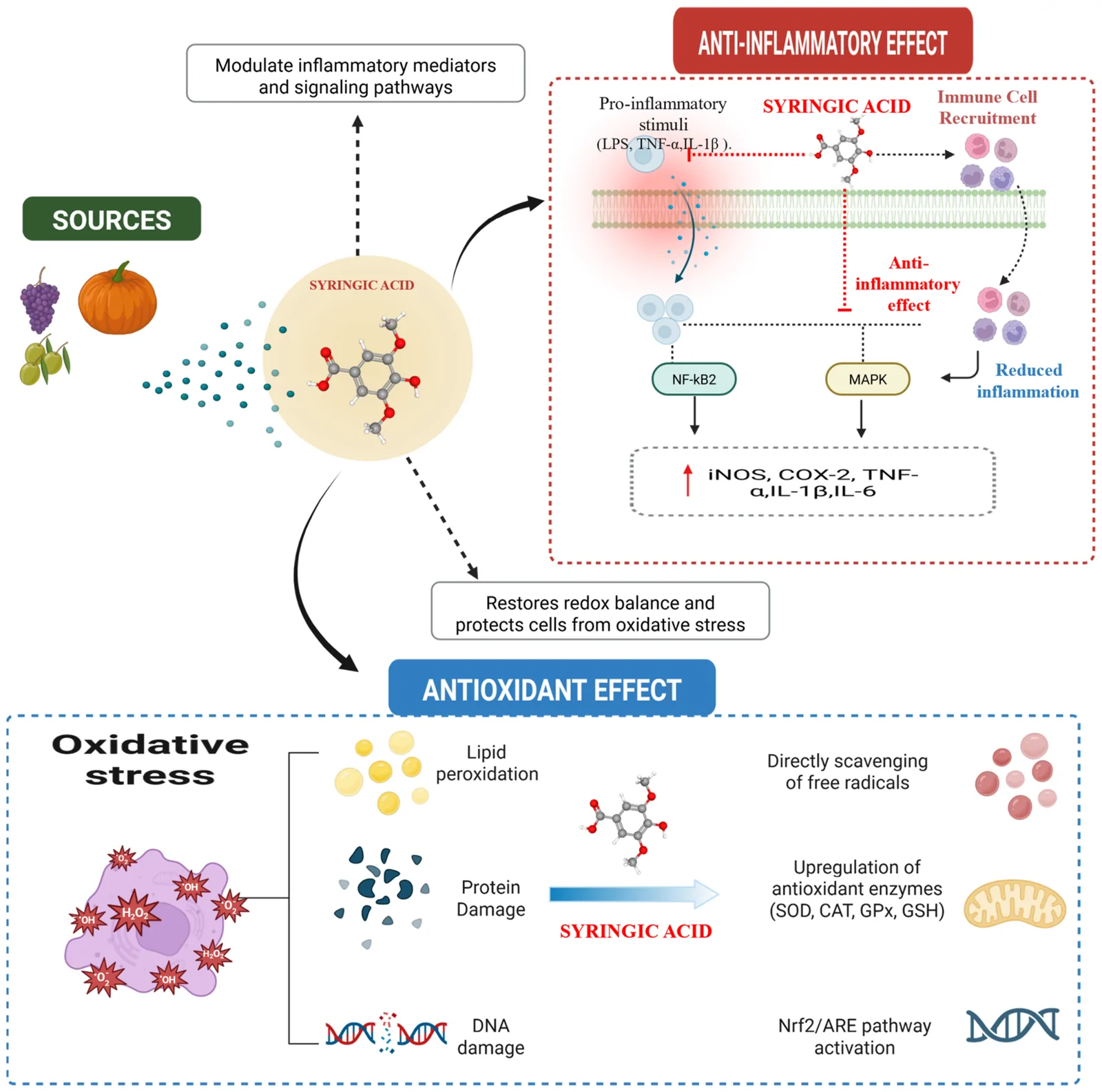
Anti-inflammatory and antioxidant mechanisms of syringic acid. Natural dietary sources such as grapes, pumpkin, and olives provide syringic acid with potent antioxidant and anti-inflammatory activities. Syringic acid modulates inflammatory signaling pathways by suppressing the activation of NF-κB and MAPK, leading to reduced expression of pro-inflammatory mediators. Simultaneously, syringic acid restores cellular redox homeostasis by directly scavenging reactive oxygen species (ROS), inhibiting lipid peroxidation, protein oxidation, and DNA damage, and enhancing endogenous antioxidant defense systems through upregulation of b antioxidant enzymes. Created with BioRender.com.

Inflammation is the immune system’s response to harmful stimuli, such as pathogens, toxic compounds, damaged cells, or irradiation [24], and acts by eliminating injurious stimuli and starting the healing process [25]. While acute inflammation is an important physiological response that supports healing and recovery, persistent or chronic inflammation can lead to the development and progression of numerous diseases, such as cancer, cardiovascular diseases, and autoimmune disorders [26,27,28]. Natural compounds, including syringic acid (SA), have anti-inflammatory potential and help ease tissue damage and inflammation [Figure 3]. Anti-inflammatory activities of syringic acid (SA) in different experimental animal models are presented in Table 1. One study aimed to examine the role and mechanism of SA in treating acne inflammation caused by *C. acnes* in vitro and in vivo. Heat-killed *C. acnes* triggered notable cell apoptosis, interleukin (IL)-1β, IL-18, IL-6, TNF-α release, and ROS production, reduced CAT and SOD activity, and upregulated the expression of proteins in HaCaT cells, including upregulating IL-1β, PPARγ, NLRP3, Nrf2, HO-1, NQO1, and caspase-1. At the same time, SA inhibited these effects by partially impairing PPARγ activation. SA also showed noteworthy improvements in ear redness, swelling, and the expression of PPARγ, IL-1β, and NLRP3 in vivo [29]. The anti-inflammatory effects of syringic acid (SA) were investigated in a methyl cellosolve (MCEL)-induced model of hepatic as well as testicular inflammation. SA administration at doses of 25 mg/kg (except for hepatic iNOS), 50 mg/kg, and 75 mg/kg meaningfully reduced the levels of inflammatory biomarkers. The findings indicate that syringic acid (SA) holds powerful anti-inflammatory activities by inhibiting MCEL-caused activation of the NF-κB and JAK/STAT signaling pathways, and in that way, reducing inflammation and protecting hepatic as well as testicular tissues from inflammatory damage [30]. The role of syringic acid in treating high-fat (HF) diet-induced pulmonary inflammation in male rats was determined. Syringic acid (SYR) reversed the HFD-induced dyslipidemia. Additionally, the changes in both enzymic and non-enzymic oxidative indicators induced by HF intake were ameliorated by SYR. After treatment with SYR, the increased levels of inflammatory cytokines (NF-κB, IL6, COX-2, and TNF-α) in the lung tissue and BALF were decreased. Additionally, exposure to HF lowered the inflammatory indices measured in BALF, whereas the levels of LDH and globulin were increased [31]. The effects of syringic acid (SA) against testicular damage in rats were analyzed. It was reported that SA administration orally dose-dependently inhibited endoplasmic reticulum stress, inflammation, oxidative stress, and apoptosis and preserved testicular histoarchitectures [32]. A study result reported that SA downregulated lipogenic genes and inflammatory genes, whereas fatty acid oxidation genes in the liver were upregulated. These results suggest that SA exerts anti-obesity, anti-inflammatory, and anti-steatotic effects through the regulation of inflammatory genes and lipid metabolism [33]. Syringic acid was examined to measure its anti-arthritic effects using adjuvant-induced arthritic rats. Outcomes revealed that administration of chronic syringic acid meaningfully reduced the paw size and arthritic index. Moreover, syringic acid significantly reduced the mRNA expression of tumor necrosis factor-α and all the other inflammatory cytokines, whereas the mRNA expression of anti-inflammatory cytokines was enhanced [34].

It was reported that, in a dextran sulfate sodium (DSS)-induced mouse model of colitis, SA meaningfully modulated the activity and expression of myeloperoxidase (MPO) and CD68^+^, a marker of macrophages, demonstrating that SA may attenuate immune cell activation and inflammatory responses [35].

### 4.2. Antioxidant Potential of SA

Oxidative stress (OS) is a biological condition that arises when there is an excess of oxidized molecules in cells, disrupting the redox balance [36]. An imbalance between free radical production and the body’s capability to detoxify them or repair the resultant damage leads to OS [37]. This imbalance can result from increased free radical production, reduced antioxidant defenses, or both [37]. While moderate levels of RNS and ROS are essential for cellular signaling and maintaining homeostasis [38], when ROS is produced in excess or when antioxidant defenses are compromised, redox homeostasis is altered, activating oxidative damage and cell dysfunction. These changes are closely related to a pathological process [39,40].

Natural compounds play a crucial role in managing oxidative stress via various mechanisms that improve antioxidant defenses as well as restore redox balance in the body [41].

Moreover, syringic acid antioxidant effects were evidenced by in vivo and in vitro studies as syringic acid enhances antioxidant enzymes levels and reduces oxidative stress [Figure 3]. Antioxidant activities of syringic acid (SA) in different experimental animal models are presented in Table 1. The antioxidant efficacy of syringic acid (SA) was assessed in a rat model of L-arginine-induced acute pancreatitis (AP). Induction of AP caused marked oxidative damage, as evidenced by significant elevations in pancreatic tissue total oxidant status, lipid hydroperoxide, and oxidative stress index, along with marked reductions in total antioxidant status and sulfhydryl (–SH) group levels compared with controls. Treatment with SA efficiently restored the oxidant–antioxidant balance by lowering oxidative stress biomarkers and enhancing antioxidant defenses. These findings demonstrate that SA protects pancreatic tissue against L-arginine-induced injury through its potent antioxidant activity and its ability to mitigate oxidative stress [5]. The role of SA against testicular toxicity in rats administered lead acetate (PbAc) was investigated. While PbAc disturbed the seminiferous tubules as well as produced atrophic images, SA corrected such histological abnormalities. PbAc administration reduced the levels of GPx, CAT, SOD, GSH, NQO1, and NRF-2 and increased the levels of 8-OHdG and MDA in the testicular tissue of rats, whereas SA improved this condition [42]. The protective role of syringic acid (SA) against testicular torsion/detorsion (T/D)-induced injury was estimated in an experimental model. T/D injury evidently increased oxidative stress, endoplasmic reticulum stress, inflammatory responses, and apoptotic compared to the control group. Treatment by SA meaningfully attenuated these alterations in a dose-dependent manner, indicating its ability to preserve testicular tissue integrity. These findings suggest that the protective effects of SA are mediated through its antioxidant, anti-inflammatory, anti-endoplasmic reticulum stress, and anti-apoptotic properties [43]. The study was conducted to evaluate the effect of SA in the streptozotocin-induced diabetic nephropathy model. It was reported that there was a rise in MDA levels and a noteworthy fall in GSH content in renal tissues of the diabetic group compared with the normal control group, demonstrating increased renal lipid peroxidation due to excess oxidative species in tissue. SA treatment caused enhanced GSH levels as well as a noteworthy decrease in MDA levels in renal tissue of hyperglycemic rats, demonstrating improved ROS scavenging under hyperglycemic conditions [44]. The study was made to evaluate the protective role of SA on isoproterenol (ISO)-induced myocardial infarction (MI). The outcomes of the study confirmed that the cardioprotective potential of SA is due to anti-lipid peroxidative as well as endogenous antioxidant system improvement effects [45]. Another study result reported that treatment by SA meaningfully reduced toxicity markers, lowered hyperglycemia, and improved insulin levels in diabetic rats. Additionally, SA increased the activity of enzymes such as catalase, superoxide dismutase, glutathione reductase, and glutathione peroxidase in the pancreas [46]. One study examined the protective role of SA in COX inhibitor-induced gastric injury. It was noted that COX inhibitor exposure produced severe mucosal damage through oxidative stress, inflammation, and apoptosis. Pretreatment with SA reduced the ulcer index and increased the gastric pH. The ulcer inhibition rates were reported as 78.5% for SA and 91.5% for OMP. SA evidently improved the tissue architecture, reduced MDA levels, restored antioxidant-enzyme activity, and downregulated NF-κB, iNOS, and COX-2. It also shifted the Bax/Bcl-2 ratio toward anti-apoptosis and lowered cleaved caspase-3 expression [47]. Another study result reported that increased levels of lipid peroxidation products, blood pressure, and renal and hepatic function markers, and the reduced levels of NOx and antioxidants in L-NAME-induced hypertensive rats, were upturned by SA treatment [48]. The effect of SA in prenatal VPA-treated rats was determined by oxidative stress, behavior, neuroinflammation, neurotransmitters, neuronal integrity, and apoptotic markers. Rats’ treatment with SA dose-dependently prevented behavioral alteration, reduced neuroinflammatory markers, restored antioxidant enzymes and neurotransmitter levels, and improved neuronal integrity [49].

**Table 1:** Anti-inflammatory and antioxidant activities of syringic acid (SA) in different experimental animal models. Overall, SA enhanced endogenous antioxidant defense systems and reduced oxidative stress markers. In inflammatory models, SA suppressed the production of pro-inflammatory cytokines and mediators. These findings demonstrate the potent antioxidants and anti-inflammatory properties of SA across diverse pathological conditions.

Activity	Study Model	Doses	Findings	Refs.
Anti-inflammatory potential	High-fat diet-induced obese mice	High-fat diet with SA (0.05%, wt/wt)	° SA reduced TNFα, IFNγ, and IL-6 and downregulated inflammatory genes	[33]
	Methyl cellosolve-induced hepato-testicular inflammation in rats	25, 50, and 75 mg/kg	° Anti-inflammatory effect of SA reflected in inhibiting the activation of the NF-κB as well as JAK-STAT signaling pathways	[30]
	Dextran sulfate sodium-induced colitis in mice	50 mg/kg	° These findings highlight the promising role of syringic acid as both a preventive and therapeutic candidate for the management of inflammatory bowel disease	[50]
	DMN-induced hepatotoxicity in rats	50 mg/kg	° SA significantly reduced NF-κB, TNF-α, and IL-1β	[51]
Antioxidant potential	Streptozotocin-induced diabetes in rats	50 mg/per kg	° Syringic acid increased the activity of antioxidant enzymes in the pancreas	[46]
	Ovalbumin (OVA)-induced asthma in mice	25 and 50 mg/kg	° Enzymatic and nonenzymatic antioxidants were high in the syringic acid treatment group	[7]
	Indomethacin-induced gastric injury in rat	50 mg/kg	° Syringic acid shows gastroprotective effects by modulating oxidative stress, inflammation, and apoptosis	[47]
	(L)-NAME-induced hypertension in rat	25, 50, and 100 mg/kg	° The increased levels of hepatic lipid peroxidation products and the reduced levels of NOx and antioxidants were reversed by the SA treatment	[48]
	Valproic acid induced autism in rats	25, 50, and 100 mg/kg	° Dose-dependently restored antioxidant enzymes and improved neuronal integrity by SA	[49]

Abbreviations: SA, syringic acid; NOx, nitric oxide metabolites; TNF-α, tumor necrosis factor-alpha; IL, interleukin; NF-κB, nuclear factor kappa B; JAK/STAT, Janus kinase/signal transducer and activator of transcription; DMN, dimethylnitrosamine.

### 4.3. Anti-Diabetic Potential of SA

Diabetes mellitus (DM) is a debilitating endocrine disease characterized by the most severe metabolic abnormalities, including hyperglycemia and its consequences [52]. Although considerable advances have been attained in diabetes management with the development of oral antidiabetic medications over the past decades, optimal glycemic control and long-term clinical outcomes remain challenging for many patients. SA demonstrated antihyperglycemic activity by improving the glycemic status, enhancing insulin secretion, reducing oxidative stress, and protecting from pancreatic and renal damage, as presented in Table 2. Oral administration of SA positively modulates the glycemic status in diabetic rats. Plasma glucose levels diminished, with a noteworthy increase in plasma insulin and C-peptide levels. The altered levels of tissue and plasma glycoprotein components were returned to near normal [53]. Another study reported that SA treatment improved insulin levels, lowered hyperglycemia, and decreased toxicity markers in diabetic rats. Additionally, SA promoted the activity of enzymes such as glutathione reductase, catalase, superoxide dismutase, and glutathione peroxidase in the pancreas [46]. Other study findings demonstrated that syringic acid (SA) efficiently ameliorated diabetes-induced biochemical aberrations by reducing most plasma biochemical parameters in diabetic rats. SA administration also attenuated oxidative stress, as evidenced by reduced hepatic malondialdehyde (MDA) levels and enhanced catalase (CAT) activity. These biochemical improvements were consistent with histopathological observations, which revealed marked restoration of tissue architecture in SA-treated animals compared with untreated diabetic controls [54]. The role of SA on diabetic nephropathy, as well as mitochondrial biogenesis, was studied. ALP and blood glucose levels were meaningfully reduced in SA-treated rats. SA increased the kidney GSH content in the diabetic group. Additionally, superoxide dismutase (SOD) and catalase levels in the diabetic group were returned toward normal levels after SA administration. Renal TBARS levels were reduced by SA, which were elevated in diabetic rats. Histopathological examination further demonstrated a noteworthy improvement in renal tissue alterations by SA treatment as compared to the diabetic group [55]. The oral administration of SA dose-dependently improved the glycemic status in diabetic rats. The levels of insulin, glycogen, and Hb increased with a decrease in glucose and HbA1c levels in SA-treated rats. The altered hepatic and renal markers and activities of carbohydrate metabolic enzymes were restored to near normal. Histopathological examination of pancreatic tissue confirmed that SA evidently alleviated alloxan-induced pancreatic damage and promoted the regeneration of pancreatic β cells in diabetic rats, indicating its protective and restorative effects on pancreatic architecture and function [56]. The protective role of syringic acid (SA) against diabetes-associated organ complications was evaluated in streptozotocin-induced neonatal diabetic rats. Oral administration of SA (25 and 50 mg/kg) meaningfully attenuated diabetes-related abnormalities, including hyperglycemia, polydipsia, polyphagia, polyuria, relative organ weights, cardiac hypertrophy, elevated biomarkers of tissue injury and inflammation, glycated hemoglobin, and oxidative stress, restored Na^+^/K^+^-ATPase activity, and markedly improved histopathological alterations. These findings advocate that SA exerts a broad protective role against diabetic complications by mitigating oxidative stress as well as inflammation, while preserving the structural integrity of multiple organs [57]. The protective effects of syringic acid (SA), isolated from *Dendrobium* species, were explored in a model of diabetic cataracts (DC). SA effectively preserved lens transparency and delayed the development of lens turbidity in D-galactose-induced cataracts by targeting AR [58].

**Table 2:** Study models, administered doses, and anti-diabetic effects of syringic acid. SA demonstrated antihyperglycemic activity by improvement of glycemic status, enhancement of insulin secretion, reduction in oxidative stress, and protection of pancreatic and renal damage.

	Study Model	Doses	Findings	Refs.
Antidiabetic effects	Alloxan-induced diabetic rat model	50 mg/kg	° SA significantly lowers plasma glucose concentrations while enhancing plasma insulin levels	[53]
	Streptozotocin-induced diabetic rat model	50 mg/kg	° SA lowered hyperglycemia and improved insulin levels	[46]
	Streptozotocin-induced diabetic rat model	25, 50 and 100 mg/kg/day	° SA decreased plasma glucose, SGOT, SGPT and TG	[54]
	Streptozotocin-induced diabetic rat model	25, 50 and 100 mg/kg/day	° SA reduced blood glucose and ALP level ° SA increased kidney GSH content, renal catalase and superoxide dismutase activities	[55]
	Alloxan-induced diabetic rat model	25, 50 and 100 mg/kg/day	° Syringic acid (SA) administered at 50 mg/kg significantly improved glycemic control ° SA treatment increased insulin, hemoglobin (Hb), and hepatic glycogen levels while significantly reducing blood glucose and glycated hemoglobin (HbA1c) concentrations ° SA attenuated pancreatic tissue injury	[56]
	Streptozotocin-induced diabetic rat model	25 and 50 mg/kg/day	° SA reduced hyperglycemia, polydipsia, polyphagia, and polyuria	[57]

Abbreviations: SA, syringic acid; ALP, alkaline phosphatase; GSH, reduced glutathione; Hb, hemoglobin; HbA1c, glycated hemoglobin; SGOT, serum glutamic–oxaloacetic transaminase; SGPT, serum glutamic–pyruvic transaminase; TG, triglycerides.

### 4.4. Cardioprotective Effects of SA

**Figure 4:**
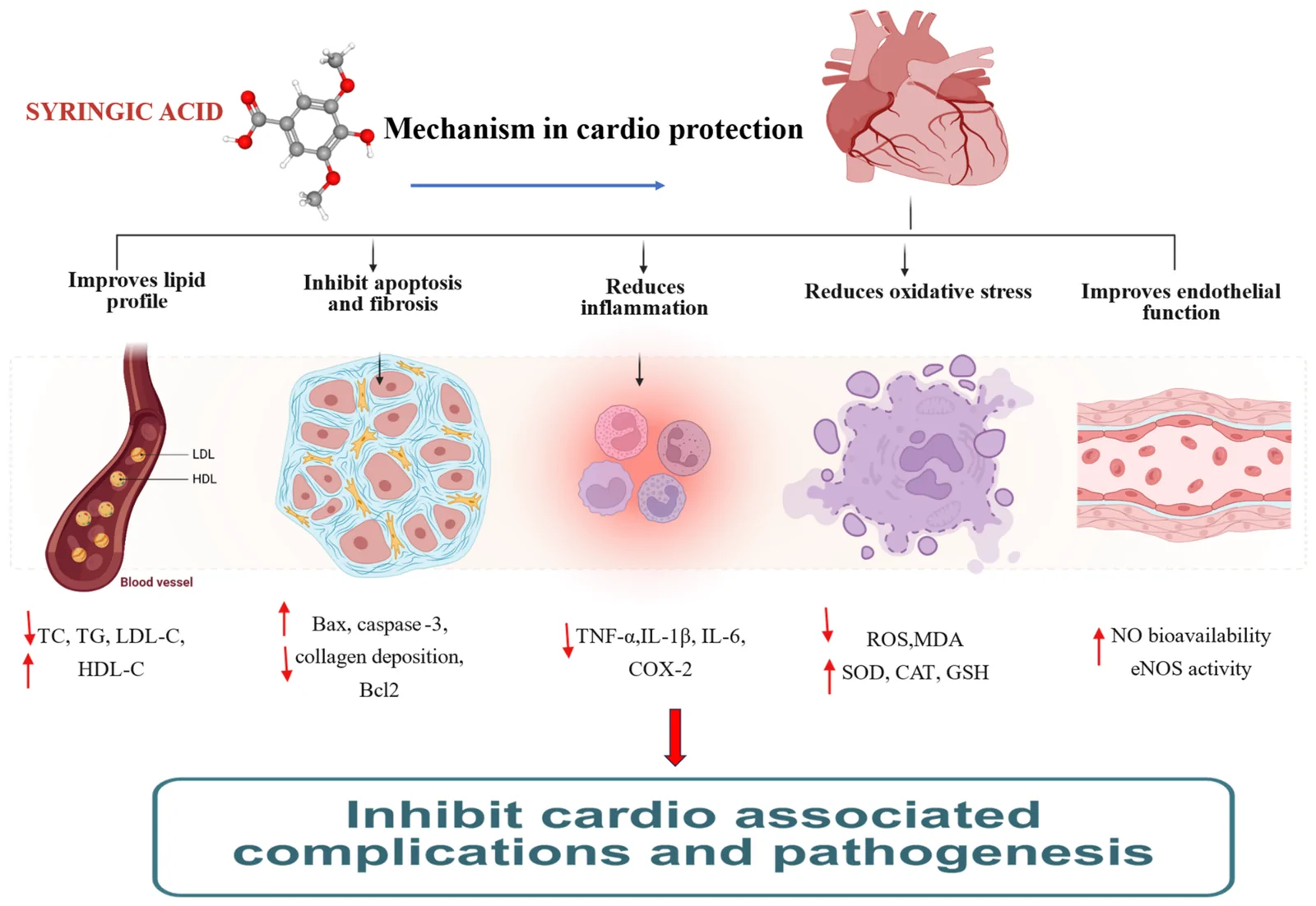
Cardioprotective mechanisms of syringic acid. Syringic acid exerts cardioprotective effects through multiple mechanisms that collectively preserve cardiac structure and function. It improves lipid metabolism, inhibits cardiac fibrosis and suppresses inflammatory responses. Created with BioRender.com.

Syringic acid exerts cardioprotective effects mainly through its antioxidant and anti-inflammatory activities. It inhibits inflammation and reduces cardiomyocyte injury and cell death. Overall, these actions may protect cardiac tissue from oxidative, inflammatory, and ischemic damage. These are presented in Table 3 and Figure 4. The cardioprotective role of syringic acid (SA) was assessed in a rat model of isoproterenol (ISO)-induced myocardial infarction (MI). Pretreatment with SA meaningfully attenuated ISO-induced cardiac injury by reducing serum levels of marker enzymes, including creatine kinase-MB (CK-MB), lactate dehydrogenase (LDH), aspartate aminotransferase (AST), and alanine aminotransferase (ALT). SA also inhibited lipid peroxidation, protein carbonyl formation, as well as the production of pro-inflammatory cytokines, while enhancing the activities of antioxidant enzymes and GSH levels. Furthermore, SA improved the erythrocyte morphology and decreased the infarct size. These biochemical results were further supported by histopathological findings, and the protective effects of SA were revealed to be dose-dependent [45]. Another study reported that lactate dehydrogenase (LDH), creatine kinase-MB (CK-MB), and gamma-glutamyl transferase (GGT) levels, as well as high-sensitivity C-reactive protein (hs-CRP), were increased in serum, whereas they were reduced in the hearts of the ISO group of rats. CAT and SOD were found to be reduced significantly, while TNF-α and NF-kB expression levels were increased in the heart tissues of ISO-administered rats. Body weights decreased, and heart weights increased in the ISO group of rats. Post-treatment with SA recovered myocardial damage caused by administration of ISO [59].

Moreover, it was reported that SA alleviated the ISO-induced upregulation of heart weight-to-body weight ratio, pathological cardiac remodeling, as well as fibrosis in mice. SA evidently downregulated fibrosis-related factors and collagen accumulation. SA or transfection with si-Ereg mitigated the isoproterenol-induced upregulation of Ereg, Ngfr, and Myc. Mechanistically, SA alleviated cardiac fibrosis and hypertrophy by downregulating Ereg [60]. The cardioprotective effects of the combination (COMB) of syringic acid (SA) and resveratrol (RV) were further demonstrated in an isoproterenol (ISO)-induced myocardial injury model. ISO administration caused noticeable elevations in creatine kinase-MB (CK-MB), LDH, and ALP, while meaningfully reducing cardiac tissue CK-MB, LDH, SOD, as well as catalase activities. In addition, ISO induction increased triglycerides, total cholesterol, low-density lipoprotein cholesterol, very-low-density lipoprotein cholesterol, and TBARS, beside a reduction in high-density lipoprotein cholesterol in the serum and heart. Pretreatment with COMB significantly reversed the ISO actions [61]. The antihypertensive effect of syringic acid (SA) was examined in L-NAME-induced hypertensive rats. Administration of L-NAME significantly raised the blood pressure, impaired hepatic and renal function markers, increased lipid peroxidation, and reduced antioxidant defenses and NOx. Treatment with SA effectively ameliorated these pathological alterations by lowering blood pressure, restoring liver and kidney function markers, and enhancing antioxidants and NO. Histopathological analyses further corroborated the biochemical findings [48]. Pretreatment with syringic acid can mitigate myocardial ischemia–reperfusion injury by inhibiting mitochondria-induced apoptosis, which is regulated by the PI3K/Akt/GSK-3β signaling pathway [62]. A study examined the protective effect of syringic acid (SA) against cyclosporine A (CsA)-induced cardiotoxicity in mice. The findings revealed that CsA significantly increased the serum activities of aspartate aminotransferase (AST), lactate dehydrogenase (LDH), and creatine kinase-MB (CK-MB), along with elevated troponin I levels, indicating substantial cardiac injury. Moreover, CsA activated oxidative stress by raising the levels of cardiac oxidants, NO, and MDA, and reducing the activities of cardiac antioxidants. Additionally, CsA meaningfully increased the protein expression of cardiac pro-inflammatory cytokines, IL-1β and TNF-α, and provoked cardiac apoptosis. The histopathological evaluations confirmed the above-mentioned results. However, all these changes were significantly mitigated in SA-treated mice [63].

**Table 3:** Cardioprotective role of syringic acid by reducing oxidative stress, inflammation, and pathological cardiac remodeling, while improving myocardial function and overall cardiovascular integrity in a dose-dependent manner.

	Study Model	Doses	Findings	Refs.
Cardioprotective effects	Isoproterenol-induced cardiotoxicity in rats	12.5, 25 and 50 mg/kg	° Myocardial damage was averted by pre-co-treatment of SA	[45]
	Isoproterenol-induced post-myocardial toxicity in rats	50 mg/kg	° SA exhibited recovery of myocardial damage	[59]
	Isoproterenol-induced cardiac hypertrophy and fibrosis in mice	100 mg/kg/day	° SA upregulates heart weight to bodyweight ratio ° Pathological cardiac remodeling and fibrosis decreased	[60]
	l-NAME-induced hypertension in rats	25, 50 and 100 mg/kg/day	° SA decreased oxidative stress and retained NO bioavailability	[48]
	Myocardial ischemia reperfusion injury rat	25, 50,75 and 100 mg/kg/day	° SA mitigates myocardial ischemia reperfusion injury	[62]
	CsA-induced cardiotoxicity in mice	25, 50 and 100 mg/kg/day	° SA modulates oxidative stress, apoptosis and inflammation	[63]

Abbreviations: SA, syringic acid; CsA, cyclosporine A; NO, nitric oxide.

### 4.5. Neuroprotective Effects of SA

**Figure 5:**
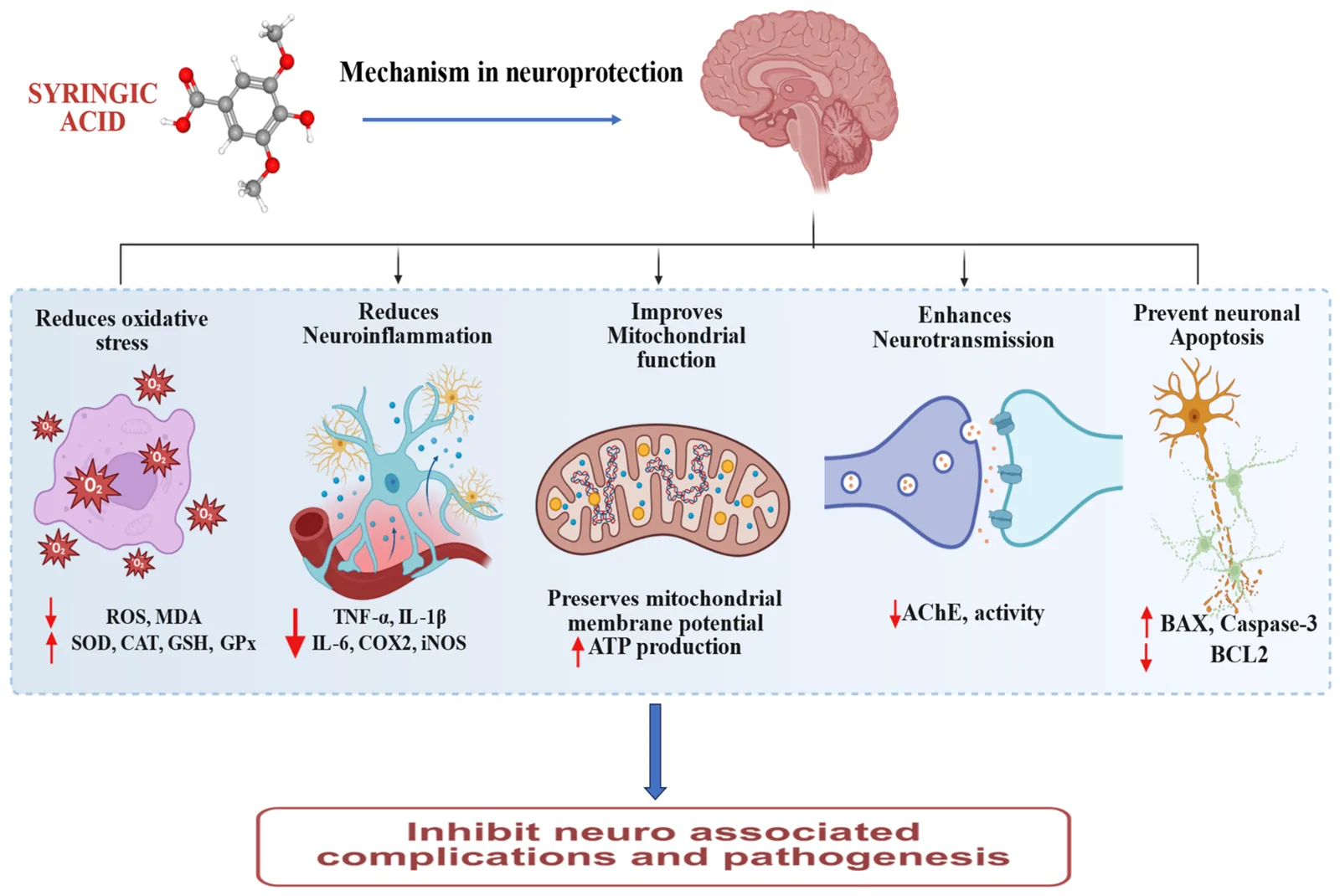
Neuroprotective mechanisms of syringic acid. Syringic acid exerts neuroprotective effects through multiple mechanisms that preserve neuronal structure and function. It attenuates oxidative stress and enhances endogenous antioxidant defenses. Syringic acid suppresses neuroinflammation by decreasing the production of pro-inflammatory mediators. Furthermore, it preserves mitochondrial integrity by maintaining mitochondrial membrane potential and enhancing ATP production. Created with BioRender.com.

Syringic acid exerts neuroprotective effects mainly through its antioxidant and anti-inflammatory activities. It increased antioxidant enzymes, thereby reducing oxidative stress. It can also inhibit inflammation and reduce nerve cells’ injury and cell death [Figure 5]. Overall, these actions may protect nerves from oxidative, inflammatory, and ischemic damage, as presented in Table 4. A study result reported that Alzheimer’s disease (AD) rats showed learning impairments and reduced memory, augmented short-term memory loss, as well as diminished locomotor activity. It was noted that supplementation of SA to the AD rats stabilized the neurobehavioral impairments [64]. Another study reported that traumatized rats, followed by treatment with SA (25 and 50 mg/kg, per oral) for 14 days, had significantly improved memory performance. Further, SA treatment meaningfully attenuated increased AChE activity and TNF-α levels, restored antioxidant levels, and recovered mitochondrial capacities. The outcomes of this study show that the therapeutic role of syringic acid involves inhibiting inflammatory reactions against brain injury-induced cognitive dysfunction and neuroinflammation in rats [65]. A study confirmed that ischemia significantly reduced levels of nuclear respiratory factor 1 and the antioxidant enzyme superoxide dismutase (SOD), whereas SA treatment increased them. Conversely, the ischemia-induced elevation in malondialdehyde was evidently attenuated following SA administration. Furthermore, SA reduced the caspase-3 and caspase-9 values. Overall, these findings show that SA exerts neuroprotective effects by alleviating oxidative stress, suppressing apoptosis, and reducing neuronal degeneration [66]. Other study results demonstrated that pre-oral treatment with SA improves MPTP/p-induced motor impairment by restoring antioxidant enzyme levels and the catecholamine content. In addition, it ameliorated the expression of tyrosine hydroxylase, vesicular monoamine transporter-2, and dopamine transporter, and also attenuated MPTP/p-induced increases in inflammatory marker expression [67].

The study assessed the neuroprotective role of SA on oxidative damage as well as mitochondria in the brains, sciatic nerves and spinal cords of streptozotocin-induced diabetic rats. It was noted that diabetic rats treated with 100 mg/kg SA showed meaningfully improved learning, movement deficiency and memory. SA suggestively upregulated the brain mRNA expression of NRF-1 and PGC-1*α*, oxidative phosphorylation, and mitochondrial biogenesis. Furthermore, SA at 50 and 100 mg/kg increased the mtDNA/nDNA ratio in the spinal cords and brains of diabetic rats. SYR reduced lipid peroxidation in all the tissues [68]. A study assessed the neuroprotective role of SA in a 6-hydroxydopamine (6-OHDA)-induced rat model of Parkinson’s disease (PD). The outcomes confirmed that SA evidently ameliorated 6-OHDA-induced motor dysfunction, suppressed the overexpression of iNOS, restored nigral dopamine release, and diminished elevated nitrite/nitrate levels. Additionally, SA reduced the loss of nigral TH-positive cells, whereas total antioxidant (TAS) capacity increased and TOS capacity decreased in the substantia nigra (SN) of PD rats [69]. Irmak Ferah Okkay et al. 2022 [70] stated that syringic acid (SA) effectively reduced inflammatory injury by suppressing the expression of the pro-inflammatory mediators TNF-α, NF-κB, and IL-1β, while meaningfully increasing the anti-inflammatory cytokine IL-10. SA also decreased the apoptotic marker caspase-3 expression in both brain and liver tissues. Immunohistochemical investigations further confirmed that SA downregulated the expression of iNOS, GFAP, and 8-OHdG. SA reduced the deteriorating effects of thioacetamide, as noted by preserved structural integrity of hepatocytes and astrocytes and reduced ammonia concentration. Behavioral assessments, including the elevated plus maze and open-field locomotor tests, further confirmed that SA suggestively improved thioacetamide-induced behavioral deficits [70]. It was reported that scopolamine (SCO) reduced postsynaptic density protein 95 (PSD-95) expression, whereas GSK-3β, TNF-α, and caspase-3 expression increased. SA treatment decreased GSK-3β, TNF-α, and caspase-3 expression and increased PSD-95 expression. Compared with the control group, the number of hippocampal neurons was reduced in the Alzheimer’s group, but the number of neurons in the SA group was higher than in the Alzheimer’s group [71]. Rats treated with SA dose-dependently prevented behavioral alteration, decreased neuroinflammatory markers, restored antioxidant enzymes and neurotransmitter levels, and improved neuronal integrity. Furthermore, SA reduced the expression of the p38 MAPK marker in VPA-induced autistic behavior [49]. Another study confirmed that syringic acid (SA) suggestively alleviated diabetic complications and protected against renal, cardiac, neuronal as well as hepatic injury in neonatal streptozotocin (nSTZ)-induced diabetic rats [57].

**Table 4:** Neuroprotective effects of syringic acid by improving cognitive and motor functions, attenuating oxidative stress, reducing neuroinflammation, inhibiting acetylcholinesterase activity, restoring neurotransmitter balance, and preserving neuronal and astrocytic integrity.

	Study Model	Doses	Findings	Refs.
Neuroprotective effects	Alzheimer’s disease in rats	25 and 50 mg/kg	° Neurobehavioral impairments stabilized by SA supplementation	[64]
	Traumatic brain injury-induced cognitive dysfunction in rats	25 and 50 mg/kg	° SA improved memory performance ° SA treatment attenuated increased AChE activity and TNF-α levels	[65]
	Ischemia injury in rat brain	10 mg/kg/day	° SA treatment reduced oxidative stress and neuronal degeneration	[66]
	MPTP/p-induced Parkinson’s disease in mice	20 mg/kg/day	° SA improved impaired motor functions	[67]
	6-OHDA-induced Parkinson’s disease in rats	20 mg/kg/day	° Motor dysfunction, increased iNOS expression, and impaired nigral dopamine release restored by SA treatment	[69]
	Thioacetamide-induced encephalopathy in rats	50 and 100 mg/kg/day	° SA decreased the deteriorating effects and preserved astrocytes ° SA reverse behavioral impairments	[70]
	Scopolamine-induced AD-like condition in rats	50 mg/kg	° SA mitigated cognitive impairment and decreased neuroinflammation	[71]
	Valproic acid-induced autism in rat	25, 50, and 100 mg/kg	° SA prevented behavioral alteration and decreased neuroinflammatory markers ° SA restored neurotransmitter level, and neuronal integrity improved	[49]

Abbreviations: SA, syringic acid; AD, Alzheimer’s disease; AChE, acetylcholinesterase; TNF-α, tumor necrosis factor alpha; iNOS, inducible nitric oxide synthase.

### 4.6. Hepatoprotective Effects of SA

**Figure 6:**
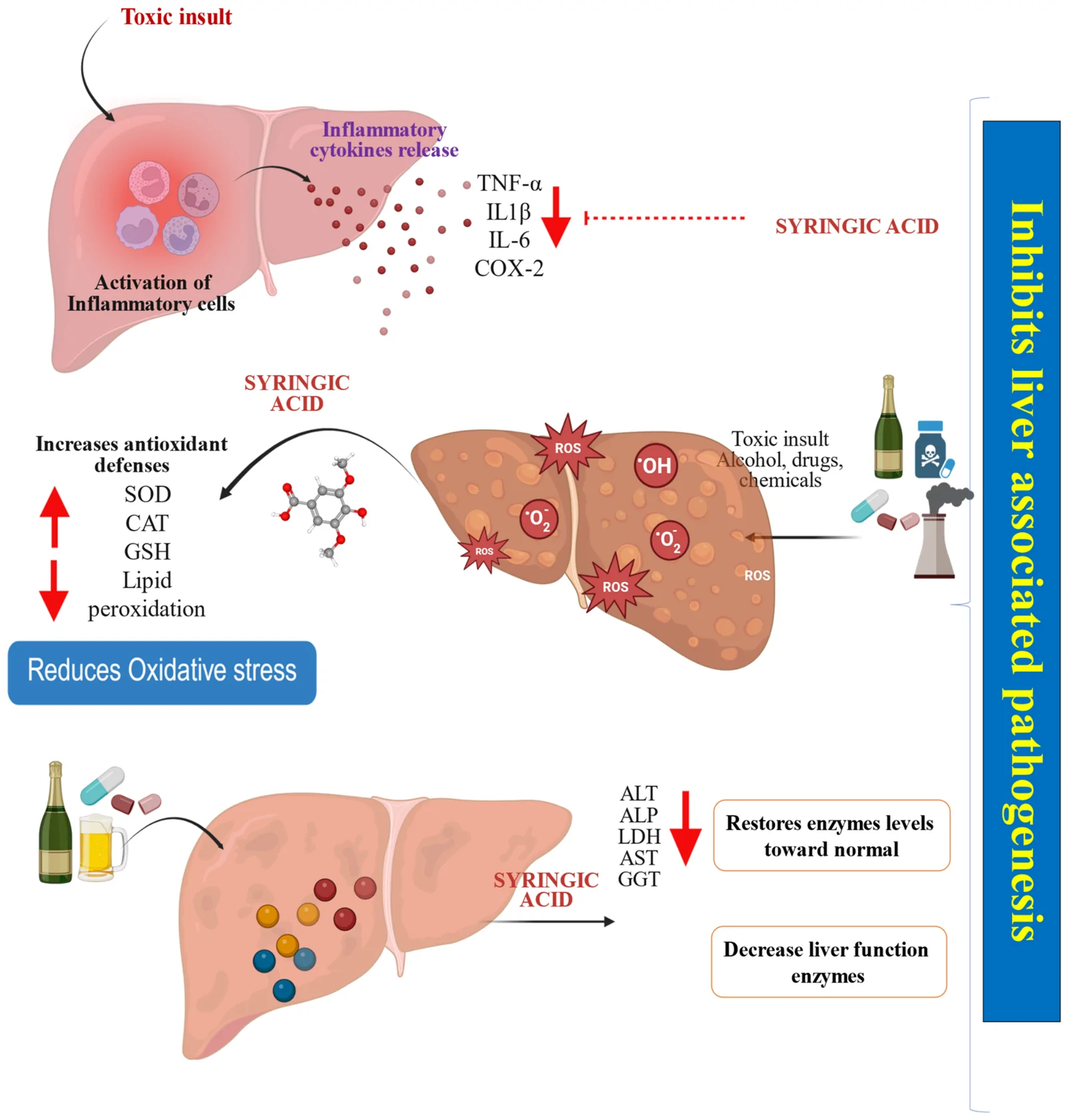
Hepatoprotective mechanisms of syringic acid. Syringic acid protects the liver against injury induced by toxic insults, including alcohol, drugs, and environmental chemicals, through multiple mechanisms. It suppresses hepatic inflammation by inhibiting inflammatory cell activation and reducing the production of pro-inflammatory mediators. Syringic acid also attenuates oxidative stress by scavenging reactive oxygen species (ROS), decreasing lipid peroxidation, and enhancing endogenous antioxidant defenses. Furthermore, syringic acid preserves hepatocyte integrity, thereby restoring liver function and reducing serum levels of hepatic injury biomarkers. Collectively, these mechanisms protect against liver injury and inhibit the progression of hepatic diseases. Created with BioRender.com.

As a natural compound, syringic acid has demonstrated significant hepatoprotective effects by regulating inflammatory responses, enhancing antioxidant defense mechanisms, modulating liver function enzymes, and preserving the structural and functional integrity of hepatic tissue [72,73,74,75]. Syringic acid may exert a hepatoprotective role by reducing elevated liver enzymes, meaning decreased hepatocellular injury [Figure 6]. Its antioxidant activity reduces lipid peroxidation and oxidative stress, while enhancing endogenous antioxidant defenses. These effects prevent hepatocyte damage and preserve liver tissue. Syringic acid’s hepatoprotective role is described in Table 5.

A study examined the hepatoprotective role of syringic acid (SA) against ethanol-induced liver injury. The results demonstrated that ethanol evidently upregulated hepatic expression of cytochrome P450 2E1 (CYP2E1), p47phox, gp91phox, nuclear factor kappa B, and COX-2, whereas post-treatment with SA significantly suppressed the expression of these molecules. Ethanol also reduced the expression of Nrf2, while cinnamic acid (CA) or SA restored Nrf2 levels. Also, SA markedly decreased ethanol-induced production of ROS, oxidized glutathione, IL-6, TNF-α, nitric oxide, and PGE_2_ [76]. Another study confirmed that SA treatment improved serum biochemical and lipid profiles in rats. SA markedly reduced the activities of serum marker enzymes and lowered VLDL-C as well as LDL-C levels, while increasing HDL-C and phospholipids. Also, SA significantly decreased the levels of free fatty acids, triglycerides, total cholesterol as well as phospholipids in plasma in liver and kidney tissues [77]. Sodium valproate (SV) administration caused elevations in serum liver transaminases and ALP. SV administration upregulated the expression of pro-inflammatory markers in liver tissue. Histopathological results showed hepatocellular necrosis and inflammatory cell infiltration following administration of SV. At both doses, simultaneous administration of SA and silymarin inhibited serum liver transaminase activity, oxidative stress, and the expression of pro-inflammatory markers in liver tissue [78]. The study was made to examine the effects of SA on sodium arsenite-induced hepatotoxicity. It was reported that SA, before sodium arsenite, reduced levels of liver enzymes, FBS, GTT, NO, TBARS, and TNF-α, and raised levels of total thiol, SOD, CAT, GPx, and caspase-3 expression as compared to the sodium arsenite group in mice [79]. Another study confirmed that syringic acid (SA) and vanillic acid caused important hepatoprotective and antifibrotic effects. Treatment with SA noticeably reduced transaminase levels, indicating decreased hepatocellular injury. CCl_4_ exposure for four weeks led to extensive collagen deposition, whereas SA meaningfully suppressed collagen accumulation and the hepatic hydroxyproline content. These findings suggest that SA protects against CCl_4_-induced hepatic fibrosis and maintains liver cell integrity [80]. It was reported that acetaminophen (APAP)-induced rats showed a rise in the activities of plasma hepatic markers and the level of lipid peroxidative markers. The toxic effect of APAP was also measured by means of determining the activity of enzymatic antioxidants and the nonenzymatic antioxidants decreased in APAP rats. An increase in the levels of total cholesterol, triglycerides, phospholipids, and free fatty acids in the plasma was noted in APAP-treated rats. Liver histology also exhibited convincing evidence about their protective nature against fatty changes induced during APAP intoxication. Among the two phenolic compounds, SA exhibited higher activity than VA [81]. Another study reported that SA improved lipid profiles in HFD-fed rats. SA reduced the serum alanine aminotransferase and aspartate aminotransferase levels. SA attenuated histopathological and immunohistochemical changes and suppressed pro-inflammatory cytokines in HFD-fed rats. SA reversed oxidative stress by replenishing the nonenzymatic and enzymatic antioxidant activities and suppressing malondialdehyde formation. The ameliorating properties of SA on NAFLD induced by an HFD in rats were prominent through the reversal of inflammation and oxidative stress, controlled by an intrinsic mechanism of defense against oxidative stress, the Nrf2/HO-1 pathway [82]. The study examined the therapeutic role of SA in DOX-induced hepatic injury. The results confirmed that by reducing oxidative stress and inflammation and reversing biochemical changes, syringic acid therapy substantially reduced hepatotoxicity in rats [9].

A study assessed the therapeutic effect of SA and ascorbic acid against DMN-caused hepatic damage in rats. The findings confirmed that treatment by SA and ASCA significantly ameliorated liver injury by normalizing the activities of hepatic enzymes, including alanine aminotransferase (ALT) and aspartate aminotransferase (AST). Furthermore, both treatments notably reduced malondialdehyde (MDA) levels and suppressed the production of the pro-inflammatory mediators TNF-α, IL-1β, and NF-κB, highlighting their strong antioxidant and anti-inflammatory hepatoprotective effects. Overall, both treatments were therapeutic; however, SA showed a superior protective effect compared with ascorbic acid [51].

### 4.7. Anticancer Potential of SA

**Figure 7:**
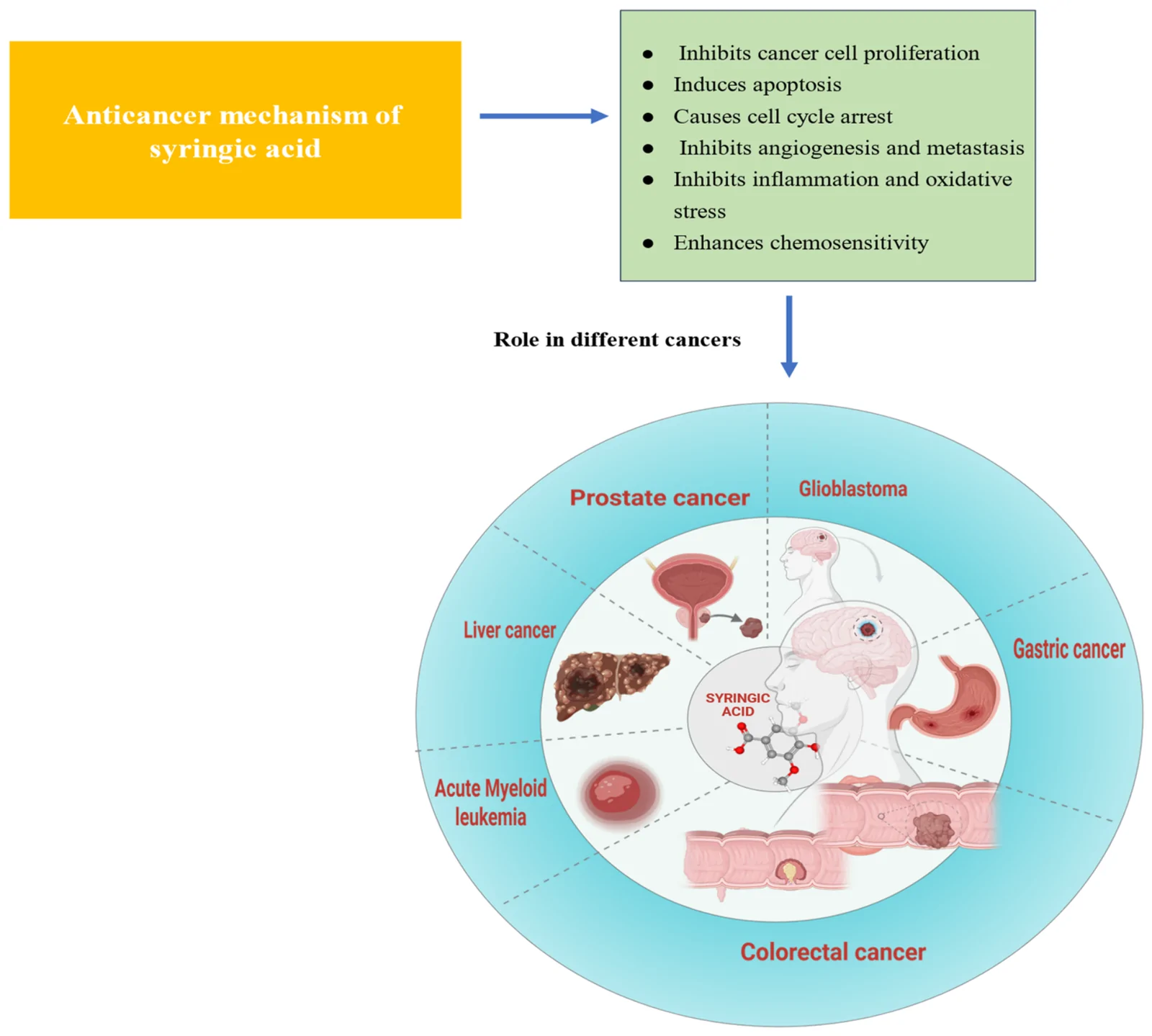
Anticancer mechanisms and therapeutic potential of syringic acid. Syringic acid exhibits broad-spectrum anticancer activity through multiple molecular mechanisms that collectively suppress tumor initiation and progression. Experimental studies have demonstrated the antitumor activity of syringic acid in several malignancies, including prostate cancer, glioblastoma, gastric cancer, oral squamous cell carcinoma, colorectal cancer, acute myeloid leukemia, and hepatocellular cancer. These diverse pharmacological actions highlight the therapeutic potential of syringic acid as a promising natural compound for cancer prevention and adjunctive cancer therapy. Created with BioRender.com.

Phenolic acids play a significant role in cancer management, as evidenced by preclinical studies, including modulation of cell signaling pathways, inhibition of cell proliferation and angiogenesis, induction of apoptosis and autophagy, cell cycle arrest, and regulation of other molecular and cellular pathways [83,84,85]. The role of syringic acid in cancer management has been established through the inhibition and activation of cellular and molecular pathways [Figure 7]. This section discusses the role of syringic acid based on in vitro and in vivo studies, as presented in Table 6.

A study on gastric cancer reported that syringic acid treatment exhibited notable anticancer activity, characterized by increased intracellular ROS, MMP loss, and reduced cell viability. Apoptosis was induced in a dose-dependent manner, accompanied by increased expression of PARP, caspase-3, and caspase-9, alongside decreased levels of BCL-2 and p53. This compound further inhibited cell proliferation and inflammation and promoted apoptosis by activating the AKT/mTOR signaling pathway. Together, these results suggest that syringic acid shows significant therapeutic potential as a chemotherapeutic candidate in gastric cancer management [86].

An experiment was conducted to examine the effects of SA on HEK 293 and HepG2 cells in the absence as well as the presence of exogenous Cu (II) and Fe(II) ions. Based on the findings, the study concluded that the combination of SA with Cu (II) was toxic to cancer cells and exhibited pro-oxidant activity. Furthermore, the combination induced autophagy in cancer cells while it caused less cytotoxic effects on normal cells, showing a potential candidate for the development of innovative therapeutics on the road to cancer [87]. The anticancer potential of SA on human cancer glioblastoma (GBM) cells was investigated. The MTT assay as well as the live/dead assay exhibited the anti-proliferative activity of SA on U-251 glioma cells. Apoptotic activity of SA was observed, and cell cycle regulation was achieved by reducing mRNA expression of cyclin D1, CDK4, and CDK6. SA treatment with U-251 cells caused and enhanced TIMP protein levels and suppressed MMP expression. It was concluded that SA prevents GBM cells’ invasion and relocation [88].

The study investigated the potential ameliorative role of SA for oxidative stress in patients with acute myeloid leukemia (AML). SA administration meaningfully increased glutathione peroxidase (GPX) activity in PBMCs. Moreover, treating PBMCs and plasma samples from AML patients with SA restored the disrupted levels of GPX, CAT and SOD. Collectively, these findings suggest that SA provides strong protection against oxidative stress by enhancing the total antioxidant status [89].

Another study’s results demonstrate that SA inhibited the proliferation of human oral squamous cell carcinoma (SCC-25) cells and induced cytotoxicity in a concentration-dependent way. Furthermore, SA treatment induced morphological features characteristic of apoptosis. These effects were accompanied by increased expression of cytochrome c, caspase-3, and caspase-9, along with an altered balance between Bcl-2 and Bax [90]. The antiproliferative and antitumor activities of SA on DU-145 cells were examined. SA meaningfully suppressed DU-145 cell proliferation in vitro. Moreover, while it decreases SOD levels, it causes a substantial increase in MDA levels. This finding revealed the antitumor activity for prostate cancer by targeting the curative SA effect [91].

The study was designed to assess the effects of SA based on in vitro and in vivo models of colorectal cancer. SA treatment resulted in substantial dose-dependent inhibition of cellular proliferation, induction of apoptosis by increasing cellular ROS and DNA damage levels, and downregulation of major proliferative genes. In vivo, SA treatment meaningfully reduced both tumor incidence and tumor volume in rats [8]. A study explored the therapeutic role of syringic acid (SA) in a diethylnitrosamine (DEN)-induced hepatocellular carcinoma rat model and explored its interaction with apoptosis-related proteins. The findings demonstrated that SA meaningfully reduced serum liver injury markers, despite increased expression of pro-apoptotic proteins in DEN-treated rats. Furthermore, SA exhibited a pronounced protective effect against DEN-induced hepatocarcinogenesis. Molecular docking analysis also supported its anticancer potential [92]. The antimitogenic and chemosensitizing effects of SA were evaluated in human colorectal cancer cells. SA exhibited a time- and dose-dependent antimitogenic effect against cancer cells. It induced cell cycle alterations in a time-dependent manner and promoted apoptosis. Notably, SA enhanced the sensitivity of cancer cells to conventional chemotherapeutic agents, significantly increasing their responsiveness to camptothecin, 5-fluorouracil (5-FU), doxorubicin, paclitaxel, vinblastine, vincristine, and amsacrine compared to treatment with these drugs alone [93].

A study on endometrial cancer demonstrated that SA meaningfully inhibited the proliferation of RL95-2 endometrial cancer cells. Co-treatment with SA and doxorubicin (Dox) produced an additive inhibitory effect. Moreover, SA and SA with Dox induced apoptosis, downregulated cyclin D expression, and upregulated the tumor suppressor proteins p21 and p27, leading to cell cycle arrest. Furthermore, both treatments evidently suppressed cancer cell migration [94]. A study examined the chemopreventive effects of syringic acid (SA) against ultraviolet B (UVB)-induced skin carcinogenesis. The outcomes confirmed that SA significantly suppressed UVB-induced expression of COX-2, PGE_2_, MMP-1, and activator protein-1 (AP-1) activity in human epidermal keratinocytes. Furthermore, SA inhibited UVB-induced phosphorylation of MAPK and Akt signaling pathways, as well as EGFR. SA also reduced intracellular ROS generation and protein tyrosine phosphatase-κ. In vivo, pretreatment with SA meaningfully decreased the incidence of UVB-induced skin tumors in mice in a dose-dependent manner [95].

**Table 6:** Anticancer effects of syringic acid by inhibition of cell proliferation, induction of apoptosis, regulation of cell cycle progression, suppression of oncogenic signaling pathways, induction of autophagy, and enhancement of oxidative stress defense in cancer cells.

Cancer Types	Study Types	Cell Lines/Animal/Human Studies	Key Findings	Refs.
Gastric cancer	In vitro	AGS	° Anticancer effects of SA were noted via apoptosis, suppression of the mTOR/AKT signaling pathway and inhibition of inflammation	[86]
Liver cancer	In vitro	HepG2	° The combination of SA with Cu (II) was toxic to cancer cells ° This combination induced autophagy in cancer cells	[87]
Glioblastoma		U-251	° SA caused anti-proliferative activity ° Apoptotic activity of SA was shown, and cell cycle regulation was confirmed	[88]
Acute myeloid leukemia	In vivo	Human patients	° SA administration increased glutathione peroxidase activity in PBMCs ° SA restored the disrupted levels of GPX, CAT and SOD	[89]
Oral squamous carcinoma	In vitro	SCC-25	° SA inhibited proliferation and induced cytotoxicity	[90]
Prostate cancer	In vitro	DU-145	° SA significantly suppressed cell proliferation	[91]
Colorectal cancer	In vivo	Colorectal cancer rat model induced by 1,2-dimethylhydrazine	° SA treatment showed tumor volume and incidence reduction	[8]
Colorectal cancer	In vitro	SW-480	° SA treatment caused cellular proliferation inhibition and induction of apoptosis	[91]
Hepatocellular carcinoma	In vivo	Hepatocellular carcinoma rat model induced by DEN	° SA reduced the liver marker levels, and expression of apoptotic proteins increased	[92]
Colorectal cancer	In vitro	SW1116 and SW837	° SA exerted significant chemotherapeutic and chemosensitizing activity	[93]
Endometrial cancer	In vitro	RL95-2 EC	° SA inhibited cell proliferation ° SA-Dox and SA combination induced apoptosis	[94]

Abbreviations: SA, syringic acid; AGS, gastric adenocarcinoma cell line; HepG2, human hepatocellular carcinoma cell line; U-251, glioblastoma cell line; SCC-25, oral squamous carcinoma cell line; DU-145, prostate cancer cell line; SW-480/SW1116/SW837, colorectal cancer lines; RL95-2, endometrial carcinoma cell line; DEN, diethyl nitrosamine.

### 4.8. Role of SA in Lung-Associated Pathogenesis/Complications

**Figure 8:**
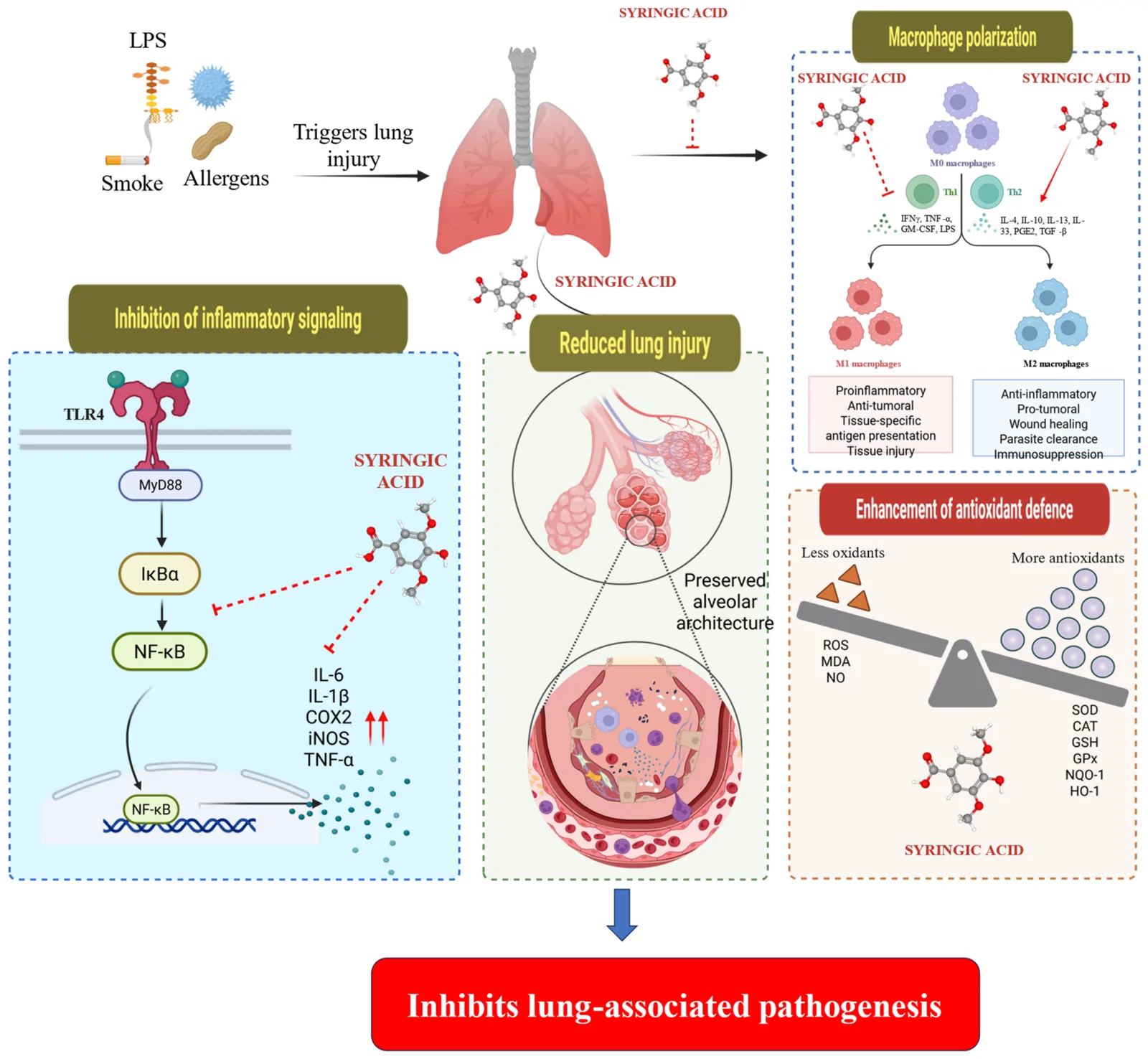
Lung-protective mechanisms of syringic acid. Exposure to toxic stimuli triggers lung injury by activating inflammatory signaling pathways, inducing oxidative stress, and impairing immune cell function. Syringic acid attenuates pulmonary injury through multiple mechanisms. It suppresses the expression of pro-inflammatory mediators. Syringic acid also modulates macrophage polarization by suppressing the pro-inflammatory M1 phenotype while promoting the anti-inflammatory M2 phenotype, thereby facilitating the resolution of inflammation and tissue repair. Collectively, these effects preserve alveolar architecture, reduce oxidative damage, pulmonary inflammation, and protect against the development and progression of lung ailments. Created with BioRender.com.

Natural compounds and bioactive ingredients have been shown to have significant therapeutic potential for regulating inflammation and reducing oxidative stress. These compounds help modulate inflammatory mediators and enhance the antioxidant defense system, in that way protecting cells from oxidative damage. In addition, they contribute to the maintenance of the lung tissue architecture and support pulmonary function [96,97]. Thus, natural compounds are increasingly being explored as auspicious agents for the prevention and management of lung-associated conditions. Syringic acids have garnered attention in recent studies for their impact on lung health and disease [Figure 8]. These compounds are known to possess anti-inflammatory and antioxidant properties that may influence the progression of various lung disorders. Experimental research indicates that phenolic acids may modulate pathways involved in lung inflammation, potentially altering disease outcomes. Furthermore, their presence in dietary sources suggests a possible protective effect against lung-related diseases. Understanding the mechanisms by which syringic acids exert their effects can lead to novel therapeutic approaches for treating lung conditions. Protective effects of syringic acid in lung-associated pathogenesis are reported in Table 7. The protective mechanism of SA against acute lung injury (ALI) was investigated, focusing on its effects on macrophage polarization and the activation of the NF-κB signaling pathway. It was observed that SA pre-treatment reduced lung injury, alveolar space, and neutrophil infiltration in the lung interstitium. SA could inhibit the production of IL-6, TNF-α, and IL-1β. Simultaneously, SA inhibited the expression of COX-2 and iNOS proteins, and the phosphorylation of IκBα and p65 proteins, in a dose-dependent manner. In vitro results proved that SA suppressed the polarization of M0 macrophages toward the pro-inflammatory M1 phenotype while facilitating the polarization of M1 macrophages toward the anti-inflammatory M2 phenotype. These outcomes indicate that SA exerts a protective effect against LPS-induced acute lung injury (ALI) in mice [98]. SA was evaluated for its ability to suppress inflammatory markers and exert anti-asthmatic effects in an ovalbumen-induced asthmatic mouse model. As a result, the SA treatment suppressed the inflammatory cells in BALF. Similarly, IgE levels were reduced in SA-treated mouse groups in the serum. In this regard, IFN-γ levels were higher in BALF of SA-treated asthmatic mouse groups, demonstrating an anti-inflammatory response. Nonenzymatic antioxidants and enzymatic levels were higher in SA treatment than in the asthmatic control group, and airway hyperreactivity was comparatively normal in the SA treatment [7]. The therapeutic potential of SA was assessed in a high-fat (HF) diet-induced pulmonary inflammation model. SA at 50 mg/kg reversed the HFD-induced dyslipidemia. The increased levels of inflammatory cytokines measured in lung and BALF tissue decreased after SA treatment. Also, exposure to HF reduced the inflammatory index (albumin) measured in BALF, whereas LDH and globulin levels increased. Also, inflammatory indices in BALF were lowered with SA treatments, while a twofold increase in the albumin/globulin ratio was noted at 50 mg/kg SA. SA confirmed anti-inflammatory and antioxidant activities by inhibiting HF-induced activation of pro-inflammatory cytokines in rats [31]. The role of SA against lung fibrosis caused by BLM was investigated. The altered BALF differential cell counts, elevated lung index, hydroxyproline, MDA, and NO levels, and reductions in SOD, GSH, CAT, and GPx in the group receiving BLM, were evidence of pulmonary toxicity. Here, 50 mg/kg SA administration meaningfully reduced the changes induced by BLM. The expression of TNF-α was significantly reduced by SA when it was stimulated by BLM. The histological examination of the BLM-treated group revealed significant lung damage, and SA lessened these effects. SA significantly reduced the Szapiel score, collagen deposition, lung edema, and fibrotic alterations. It also inhibited inflammatory cell infiltration, myofibroblast infiltration, and, mainly, lymphocytes and macrophages [99].

### 4.9. Role of SA in Digestive System-Associated Pathogenesis

Phenolic acid, including syringic acid, contributes to maintaining digestive system health by reducing oxidative stress, enhancing antioxidant defenses, and regulating inflammatory pathways. It may also help preserve intestinal barrier integrity and regulate gut microbiota, thereby supporting overall gastrointestinal homeostasis. The roles of syringic acid in digestive system health maintenance and inhibition of digestive system-associated pathogenesis are presented in Table 7. The potential role of SA in a mouse model of dextran sulfate sodium-induced colitis, via modulation of the gut microbiota, was examined. The outcomes demonstrated that oral administration of SA reduced colitis symptoms, as indicated by reduced histopathology scores and a reduced disease activity index. Moreover, administration of SA enriched the abundance of *Alistipes* and *norank_f__norank_o__Gastranaerophilales* in mice, suggesting impaired gut microbiota restoration. Remarkably, it was noted that the effects of SA were similar to those of fecal microbiota transplantation in dextran sulfate sodium-induced mice. The study reveals the potential of SA as a preventive as well as a therapeutic agent for inflammatory bowel disease [50]. The study was conducted to examine the antioxidant and anti-inflammatory activities of coumaric acid and SA on acetic acid-induced colitis in rats. Treatment with 50 mg/kg SA and 150 mg/kg coumaric acid increased HO-1, Nrf2, and NQO1 mRNA expression compared with the UC group. Also, treatment with 10, 25, and 50 mg/kg of SA and 100 and 150 mg/kg of coumaric acid decreased the tissue levels of IL-1β protein and TNF-α compared to the UC group [100]. A study was conducted to examine the therapeutic potential of syringic acid (SA) against ulcerative colitis (UC). SA treatment distinctly lessened the clinical manifestations of colitis and body weight loss compared with untreated DSS-induced mice. The effects of SA in DSS-induced mice were associated with an important decrease in the expression levels of inflammatory mediators and pro-inflammatory cytokines, producing a notable amelioration of colonic architectural disruption [35]. Another study was designed to examine the protective role of SA against intestinal I/R injury. The study findings reported that SA potentially protects Caco-2 cells from OGD/R injury, and that this effect might be attributed to its anti-apoptotic and anti-inflammatory activities, and its capability to protect the function of the intestinal barrier [101]. Furthermore, a study examined the therapeutic properties of syringic acid (SA) in a rat model of ulcerative colitis (UC). UC led to a noticeable increase in oxidative stress and inflammatory responses. A histopathological study revealed extensive colonic damage, characterized by severe inflammation, edema, and necrosis. Treatment with SA significantly attenuated these pathological changes. Furthermore, SA restored the antioxidant status by enhancing CAT activity and TAC [102].

### 4.10. Role of SA in Reproductive System-Associated Complications/Pathogenesis

This phenolic compound exhibits significant antioxidant and anti-inflammatory properties, which may help protect reproductive tissues against oxidative stress and inflammatory damage. Experimental studies suggest that syringic acid can modulate reproductive function, hormonal balance, and cellular integrity, thereby potentially inhibiting reproductive system disorders. The role of SA in mitigating HgCl_2_-induced testicular damage in rats was studied. It was noted that HgCl_2_ caused a decrease in antioxidant enzymes such as CAT, SOD, GPx activity and the GSH level, and increased the MDA level, in the testicular tissue of rats. HgCl_2_ is involved in the increase in eIF2-α, CHOP, PERK, ATF-4, TNF-α, ATF-6, NF-κB, IL-1β, Bax, Apaf-1 as well as Caspase-3 mRNA expression, decrease in sperm motility, rate of abnormal sperm, as well as increase in sperm DNA fragmentation. However, oral administration of SA dose-dependently caused the inhibition of inflammation, endoplasmic reticulum stress, oxidative stress and apoptosis, and preserved testicular histoarchitectures epididymal and sperm quality [32]. The preventive role of SA on organ damage to the reproductive system caused by bilateral ovarian ischemia–reperfusion (I/R) was checked. Administration of SA led to important increases in the levels of antioxidant enzymes. Also, in the SA-administered groups, decreases were detected in OSI and TOS levels, along with decreases in MPO, TNF-α, IL-1β and MDA levels. It has been confirmed that SA has a protective role against organ damage caused by bilateral ovarian I/R in the reproductive system [103]. CIS treatment caused low serum AMH levels, histopathological alterations, and increased ERS, inflammation, OS, as well as apoptosis in ovarian tissue. However, treatments by SA ameliorated biochemical as well as histopathological alterations via activation of Nrf2 [104]. Another study result exhibited that dose-dependent treatment with SA lowered the levels of biochemical parameters such as MPO and NO, increased antioxidant parameters, and reduced the MDA level in serum as well as ovarian tissue. SA treatment suppressed the levels of luteinizing hormones, estradiol (E2), anti-mullerian hormone, and FSH as well as ovarian follicles. SA downregulated inflammatory mediators and cytokines [105]. As compared to the control group, torsion/detorsion suggestively increased endoplasmic reticulum stress, oxidative stress, inflammation, as well as apoptosis in testicular tissue. Administration of SA diminished such alterations in a dose-dependent way. Furthermore, the histopathological results were consistent with the biochemical outcomes. Overall, these findings indicate that SA represents a hopeful therapeutic candidate for mitigating testicular damage associated with torsion/detorsion [43]. As compared to the control group, methyl cellosolve (MTC) administration meaningfully reduced the number of normal and viable spermatozoa, sperm count, MCHC, hemoglobin count, MCH, serum total cholesterol (TC), as well as luteinizing hormone (LH) levels. Meanwhile, MTC exposure increased the number of abnormal spermatozoa, RBC and WBC counts, as well as the serum activities of AST, ADH GGT, LDH, and ALT. Treatment with SA at 25 mg/kg reduced RBC and WBC counts and the serum activities of GGT, ALT, and AST, while increasing serum TC levels. At 50 mg/kg, SA decreased the number of abnormal spermatozoa and the activities of ALT, ADH, AST, and LDH, while increasing the number of normal spermatozoa, MCV, MCHC, and MCH. Moreover, administration of SA at 75 mg/kg further increased serum TC levels and reduced the activities of AST, GGT, ALT, LDH, and ADH [106]. Another study result reported that lead acetate (PbAc) disrupted the seminiferous tubules and produced atrophic images, while SA corrected these histological changes. It was revealed that PbAc reduced sperm quality in rats, whereas SA positively affected sperm quality [42].

### 4.11. Anti-Arthritis Effect of SA

Syringic acid caused anti-arthritic effects by reducing oxidative stress and inflammatory signaling, resulting in reduced pro-inflammatory cytokine production. Chronic treatment with SA significantly reduced the arthritic index and paw swelling while improving behavioral responses and restoring hematological and biochemical parameters. It also eased oxidative stress and lymphoid organ weight. A decrease in serum PGE2 levels was also noted following SA administration. These results were further supported by histopathological investigation and radiographic analysis. Furthermore, molecular docking confirmed favorable binding interactions of SA with IL-1β, TNF-α, COX-2, IL-6, and IL-4, comparable to those noted with the standard [34]. Another study result reported that during in vitro experiments, the inflammatory response in chondrocytes was observed by stimulation with IL-1β. The result indicated that SA suppressed NO, TNF-α, iNOS, COX-2, and PGE2 secretion. Furthermore, SA reduced the huge IL-1β-induced phosphorylation of the PTEN/AKT/NF-kB pathway. Also, SA alleviated the cartilage degeneration in the osteoarthritis (OA) mice model in vivo [107]. It has been reported that SA showed no clear cytotoxic effects on chondrocytes while enhancing the expression of chondrogenesis-related marker genes. RNA-seq investigation additionally revealed that the extracellular matrix–receptor interaction and TGF-β signaling pathways were significantly enriched among genes upregulated by SA, with SA also transcriptionally activating Smad3. Furthermore, SA suppressed the LPS-induced pro-inflammatory cytokines’ overproduction and reduced the expression of MMP-3 and MMP-13. Notably, treatment with SA attenuated cartilage degradation in a mouse model of osteoarthritis [108].

### 4.12. Wound-Healing Activity of SA

Syringic acid was shown to have a role in wound healing by reducing oxidative stress and inflammation, and enhanced tissue repair by stimulating collagen production and re-epithelialization, which are essential for wound closure. Its antioxidants and anti-inflammatory activities help protect damaged tissues. The wound-healing potential of 2.5% and 5.0% SA was assessed in type 2 diabetic rats using an incisional wound model. SA-treated diabetic wounds exhibited a meaningfully faster rate of wound closure and epithelialization, along with an increased total protein content and hydroxyproline, compared with diabetic wounds. After 14 days of treatment, SA-treated diabetic wounds showed a notable pro-inflammatory response suppression. Treatment of SA also increased the expression of CD31 and CD68 and promoted collagen deposition. Histological investigation revealed substantial improvements in skin architecture and re-epithelialization [109].

### 4.13. Role of SA in Skin Health

Phenolic acid supports skin health through its antioxidant and anti-inflammatory roles, which help protect skin cells from oxidative stress and inflammation. It can potentially reduce oxidative damage caused by UV exposure, in that way supporting skin barrier function and cellular integrity. The effect and mechanism of SA on acne inflammation induced by *C. acnes* in vitro and in vivo were determined. It was reported that heat-killed *C. acnes* activated notable cell apoptosis, production of ROS, interleukin-1β, IL-18, IL-6, and TNF-α release, reduction in SOD as well as CAT activity, and upregulation of the expression of proteins in HaCaT cells, while upregulating IL-1β, PPARγ, NQO1, Nrf2, NLRP3, HO-1, and caspase-1. In contrast, SA suppressed these activities, at least partially by impairing PPARγ activation. In vivo, SA evidently lessened ear redness and swelling and reduced the expression of NLRP3, PPARγ, and IL-1β [29].

### 4.14. Role of SA in Eye Health/Disease

The role of SA extracted from dendrobii on diabetic cataracts was assessed. It was reported as targeting AR. SA delivered protection from D-gal-caused damage by continually maintaining lens transparency as well as delaying lens turbidity development. The study concluded that SA acts to prevent DC in rat lenses by inhibiting AR activity as well as gene expression, meaning it has the potential to be established as a novel drug for therapeutic DC management [58].

The single-molecule optics technologies, including TEM, atomic force microscopy (AFM), Raman spectroscopy, and laser scanning confocal microscopy (LSCM), were employed to monitor the role of SA in human lens epithelial cell (HLEC) biomechanics as well as the organelle structure in real time. TEM exhibited that SA improved the ultrastructure of HLECs with respect to nuclear chromatin condensation as well as reducing mitochondrial swelling and degeneration, which provided support in the preservation of HLEC integrity in the presence of glucose. AFM exhibited a reduced surface roughness as well as stiffness with SA treatment, suggesting an improved viscoelasticity of HELCs. LSCM and Raman spectrometry revealed that these changes were associated with a modification of cell liquidity and cytoskeletal structure by SA [110]. Another study examined the effect of SA treatments on the high-fat diet (HFD)-induced ocular-inflammatory response in rats. The results reported increases in the levels of NF-κB, TNF-α, iNOS, IL-6, and COX-2 in rats served high-fat diets (HFDs). SA involvement (50 mg/kg) reversed these outcomes [111]. The protective effects of SA against H_2_O_2_-induced oxidative injury were assessed in retinal ganglion cells (RGCs). SA pretreatment meaningly attenuated damage in RGC-5 cells. SA pretreatment reduced H_2_O_2_-induced production of ROS as well as the MDA content in RGC-5 cells. Moreover, SA pretreatment downregulated the pro-apoptotic protein Bax, while enhancing Bcl-2 expression as well as cleaved caspase-3 levels. Collectively, these results suggest that SA protects RGC-5 cells against apoptosis induced by H_2_O_2_ through the PI3K/Akt signaling pathway’s activation [112].

**Table 7:** Protective effects of syringic acid in pathogenesis across multiple organ systems. These findings highlight the multi-organ therapeutic potential of syringic acid through modulation of key pathological mechanisms, including inflammatory signaling, antioxidant defense systems, and cellular stress responses.

Pathogenesis	Study Model	Doses	Outcomes	Refs.
Lung associated	ALI mice model	25, 50, and 100 mg/kg	° SA reduced the degree of lung injury, and wet–dry ratio of the lung reduced ° Thickening of alveolar septum reduced by SA	[98]
	OVA-induced asthma mice model	25 and 50 mg/kg	° SA control inflammation ° Thickened columnar epithelium was reduced by SA	[7]
	High-fat (hf) diet-induced pulmonary inflammation rat model	25 and 50 mg/kg	° SA at 50 mg/kg reversed dyslipidemia ° Inflammatory indices in the BALF were lowered by SA treatments	[31]
	Bleomycin-induced pulmonary inflammation and fibrosis in rats	50 mg/kg	° SA protects the lung against pulmonary oxidative stress, inflammation as well as fibrosis	[99]
Digestive system associated	Acetic acid-induced colitis rat model	10, 25, and 50 mg/kg	° TNF-α and IL-1β protein decreased by SA	[100]
	Dextran sulfate sodium-induced experimental colitis mice model	25 mg/kg	° SA decreased the expression levels of inflammatory mediators ° SA attenuated colitis	[35]
	Ulcerative colitis rat model	10, 25 and 50 mg/kg	° SA ameliorated histopathologic effects	[102]
Reproductive system associated	HgCl_2_-induced testicular damage rat model	25 and 50 mg/kg	° SA protected against testicular oxidative damage, inflammation, and apoptosis	[32]
	Ovarian damage rat model	10 and 50 mg/kg	° SA showed a protective role against organ damage	[103]
	Cisplatin-induced ovarian injury rat model	5 and 10 mg/kg	° SA treatments ameliorated biochemical and histopathological changes	[104]
	Cyclophosphamide-induced ovarian damage rat model	5, 10 and 20 mg/kg	° SA reduced ovarian damage	[105]
	Ischemia/reperfusion-induced testicular injury in rats	50 and 100 mg/kg	° Administration of syringic acid restored the damage in a dose-dependent manner	[43]
	Testicular damage induced by lead acetate rat model	25 and 50 mg/kg	° SA administered effective protection against PbAc-induced testicular dysfunction	[42]
Associated with joints	Adjuvant-induced arthritis rat model	25, 50 and 100 mg/kg	° SA reduced the paw size and arthritic index ° Treatment by SA produced substantial recovery from oxidative stress markers	[34]
	OA mice model	25 mg/kg	° SA relieved the inflammation and cartilaginous degradation	[107]
Wound	Diabetic type 2 rats using incisional wound model	2.5% and 5.0% SA	° SA improved wound healing	[108]
Associated with eye	HFD-induced oculo-inflammatory disorder	25 and 50 mg/kg	° SA showed anti-inflammatory effect	[111]

Abbreviations: SA, syringic acid; ALI, acute lung injury; OVA, ovalbumin; BALF, bronchoalveolar lavage fluid; TNF-α, tumor necrosis factor alpha; IL-1β, interleukin-1 beta; OA, osteoarthritis; HFD, high-fat diet.

### 4.15. Antimicrobial Activity of SA

Syringic acid (SA) shows antimicrobial action against a broad range of pathogenic microorganisms. Its antimicrobial effects are chiefly attributed to disruption of microbial cell membranes and interference with cellular metabolism. SA also interferes with microbial biofilm formation, thereby reducing bacterial adhesion and colonization. In fungal species, SA can impair cellular growth. The antimicrobial activities of syringic acid (SA) against various microorganisms are explained by antibacterial, antifungal, and antibiofilm properties in Table 8. The MICs of SA against various *C. sakazakii* strains were evaluated. Furthermore, changes in intracellular ATP concentration, membrane potential, membrane integrity and intracellular pH (pHin) were determined to assess the effect of SA on the cell membrane. It was reported that the MICs of SA against all tested *C. sakazakii* strains were 5 mg/mL. SA stops bacterial growth and causes cell membrane dysfunction, as evidenced by a decreased intracellular ATP concentration, reduced pH, changes in cellular morphology, and cell membrane hyperpolarization [113].

The fungitoxicity and antimicrobial activities of the phenolics against *Ganoderma boninense* were expressed in the inhibition of radial growth of *G. boninense* on PDA, ameliorated with the different phenolics at a range of concentrations of 0.5–2.5 mg/mL. Syringic acid exhibited strong fungitoxic activity against *G. boninense* even at the lowest tested concentration of 0.5 mg/mL. Increasing the concentration to 1.0 mg/mL resulted in complete inhibition of the pathogen [114]. This study examined the synergistic antimicrobial effects of syringic acid (SA) combined with mild heat (MH) against major foodborne pathogens, including *Escherichia coli* O157:H7, *Staphylococcus aureus*, *Listeria monocytogenes*, and *Salmonella enterica* serovar Typhimurium, in both buffer and tomato juice. The combined SA–MH treatment achieved at least a 5-log reduction in all tested pathogens, demonstrating clear synergistic bactericidal activity compared with either treatment alone. The combined treatment showed a greater increase in membrane permeability and loss of metabolic activity, as indicated by greater propidium iodide uptake, decreased iodonitrotetrazolium chloride reduction, and reduced glycolytic enzyme activities. Overall, these results indicate that the combination of SA and MH effectively inactivates a broad range of foodborne pathogens through multi-target cellular damage while maintaining product quality [115]. The antibiofilm potential of two phenolic compounds, syringic acid and vanillin, was evaluated against three biofilm-producing methicillin-resistant *Staphylococcus epidermidis* strains representing different genotypes. Biofilm formation and extracellular polymeric substance (EPS) inhibition were assessed using a resazurin assay in combination with crystal violet staining and confocal laser scanning microscopy. The effects of both compounds on EPS components, including proteins, extracellular DNA, and polysaccharides, were also analyzed. Overall, syringic acid and vanillin showed similar biofilm and EPS inhibition activities on *S. epidermidis* strains, reducing biofilm formation up to 80% and EPS up to 55%, depending on the genotype of the tested strain [116]. The study assessed the in vitro antimicrobial as well as fungitoxic effects of SA, caffeic acid (CA), and 4-hydroxybenzoic acid (4-HBA), which were identified in oil palm roots. Their antimicrobial activities and antifungal activities against *Ganoderma boninense* were measured based on the inhibition of radial growth on PDA medium at concentrations ranging from 0.5 to 2.5 mg mL^−1^. Among the phenolic compounds tested, SA revealed the strongest fungitoxic activity, meaningfully suppressing the growth of *G. boninense* even at the lowest concentration of 0.5 mg mL^−1^. Increasing the concentration of SA to 1.0 mg mL^−1^ caused in a superior inhibition of pathogen growth [114]. A study was performed to isolate as well as identify the compounds accountable for antifungal activity against *Fusarium oxysporum* f. sp. *albedinis* (Foa) from Asteriscus graveolens aerial parts’ extract. The isolated compounds were recognized as methyl gallate and SA [117].

## 5. Synergistic Effect of Syringic Acid

Phenolic acids, when combined with other bioactive compounds and drugs, exhibit synergistic action by different mechanisms, including enhanced antioxidant, anti-inflammatory, and anticancer activities. Both in vivo and in vitro studies have confirmed the synergistic effects of phenolic acids when combined with other bioactive compounds and therapeutic drugs. Co-treatment of SA and other drugs/compounds demonstrated an effective synergistic agent that enhances the efficacy of therapeutic compounds through modulation of oxidative stress, inflammation, and cellular pathways, as described in Table 9. The study explored the role of syringic acid (SA) in the proliferation as well as migration of RL95-2 endometrial cancer (EC) cells, as well as its synergistic anticancer effects when combined with chemotherapeutic agents. SA meaningfully inhibited the proliferation of RL95-2 cells, whereas co-treatment of SA and doxorubicin (Dox) produced an additive inhibitory effect. Mechanistically, both SA alone as well as the SA-Dox combination induced apoptosis. Cell cycle analysis was described as decreasing cyclin D expression and increasing levels of the tumor suppressor proteins p21 and p27, resulting in cell cycle arrest. Moreover, SA and the combination treatment noticeably decreased cell migration, which was associated with the downregulation of matrix metalloproteinases (MMPs) and β-catenin [94]. The synergistic antioxidant and antidiabetic effects of a zinc (II)–syringic acid (SA) complex were assessed in diabetic rats. Following diabetes induction, the animals received the zinc–SA complex as well as its precursors, syringic acid and zinc sulfate, for four weeks at specified doses. The findings exhibited that complex formation enhanced the antihyperglycemic effects by approximately 28–36% and 37–40%, respectively, indicating a synergistic effect associated with complexation. This is credited to the modulatory action of the complex on insulin secretion and sensitivity, muscle hexokinase activity, tissue glycogen production, as well as Akt phosphorylation, thus improving glucose tolerance in diabetic rats, relative to its precursors. In parallel, the complex increased the activity of antioxidant enzymes, reducing systemic as well as tissue lipid peroxidation in the diabetic rats [118].

A study examined the role of Gigantol and syringic acid (SA) in aldose reductase (AR) activity and cataract formation. The study evaluated the synergistic anti-cataract effects of Gigantol and SA using a high-glucose- and streptozotocin-induced diabetic cataract (DC) rat model. The combination treatment with Gigantol and SA delivered protective benefits for both human lens epithelial cells (HLECs) grown in vitro as well as for cataract formation in STZ-induced rats in vivo. The observed synergy was attributed to the inhibition of AR activity, AR expression downregulation through impaired transcription, and decreased sorbitol levels. Therefore, the combined administration of Gigantol and SA improves the anti-cataract efficiency [119]. The cardioprotective role of a combined treatment comprising syringic acid (SA) and resveratrol (RV) was examined in an isoproterenol (ISO)-induced cardiotoxicity model. ISO administration caused raised serum CK-MB, LDH, as well as alkaline phosphatase levels, accompanied by reduced cardiac tissue LDH, CK-MB, superoxide dismutase, and catalase. Moreover, ISO-treated rats showed increased levels of triglycerides, total cholesterol, very-low-density lipoprotein cholesterol, low-density lipoprotein cholesterol, as well as thiobarbituric acid-reactive substances, along with high-density lipoprotein cholesterol decreases in the serum and cardiac tissue. The mRNA levels of inflammatory markers TNF-α and NF-κB were also evidently raised following ISO exposure. Remarkably, pretreatment with the SA–RV combination significantly reduced these ISO-induced alterations. The protective effects were additionally supported by transmission electron microscopy and histopathological results, which were correlated with the above biochemical parameters [61]. The pretreatment of Sweet Sorghum Bagasse (SSB) by Coriolus versicolor with synergistic SA-gallic acid supplements for boosted ligninolytic enzyme production, lignin degradation, enzymatic saccharification, and selectivity value has been studied. Supplementing gallic acid increased the MnP production, whereas supplementing SA increased the production of AAO, LiP, and laccase with greater lignin degradation and SV. The use of combined supplements significantly enhanced the production of various ligninolytic enzymes beyond the effects seen with individual supplements, due to synergistic interactions. The activities of laccase, LiP, MnP, PPO, and AAO were increased by 9.1, 14.6, 2.6, 1.9, and 2.2 times, respectively. This led to the highest levels of lignin degradation, achieving a 1.56-fold increase, and a substantial rise in sugar SV by 3.87 times. Enzymatic hydrolysis of SSB that was pretreated with these combined supplements resulted in a greater yield of fermentable sugars, approximately 2.18 times higher. Therefore, these combined supplements can be effectively utilized to enhance pretreatment processes through SSF [120]. The synergistic effect of syringic acid, 4-hydroxybenzoic acid, and caffeic acid, which are found in oil palm root, was evaluated in vitro. The phenolic compounds were tested for their ability to inhibit the radial growth of Ganoderma boninense on PDA ameliorated with various combinations of two or three phenolics at concentrations ranging from 0.5 to 2.5 mg/mL. Several combinations demonstrated a significant fungitoxic effect against G. boninense. Notably, the combination of 4-hydroxybenzoic acid, syringic acid, and caffeic acid at concentrations up to 2.5 mg/mL effectively suppressed the growth of G. boninense when compared to the control [121].

## 6. Pharmacokinetics, Limitations, Strategies to Improve Efficacies of Syringic Acid and Syringic Acid Derivatives’ Role in Disease

Pharmacokinetics is the study of how a compound is absorbed, distributed, metabolized, and eliminated by the body after administration. Understanding pharmacokinetic behavior and bioavailability is important for assessing its therapeutic role and its possibility as a drug candidate and dosage optimization. A pharmacokinetic study was performed to evaluate the absorption and bioavailability of syringic acid (SA) in rabbits. After the rabbits were injected with SA via the ear vein and the peritoneal cavity, blood samples were collected from the ear vein at predetermined time points, and SA concentrations in the blood were quantified using HPLC. Pharmacokinetic investigation showed the concentration–time profile of SA after intravenous administration in a two-compartment open model, and absolute bioavailability was 86.27% [122]. Despite its high bioavailability in rabbits, syringic acid (SA) shows poor oral absorption and rapid elimination, resulting in a short half-life (T½) and comparatively low bioactivity [123].

Existing evidence shows that the main pharmacokinetic limitations of SA are poor aqueous solubility, low oral bioavailability, and rapid elimination. These factors might partially describe the discrepancy between the promising therapeutic effects noted in preclinical studies and the restricted clinical translation of SA. Several formulation-based strategies have been investigated to overcome these limitations. This formulation improves absorption and systemic exposure when providing continued drug release. Moreover, these nanotechnology-based approaches can significantly enhance the various pharmacological activities through modulation of biological activities.

Here, syringic acid-based nanoformulations and their roles in preclinical studies are discussed. The SA-loaded TPGS liposome (SA-TPGS-Ls) was prepared to improve the oral bioavailability of SA. SA-TPGS-Ls exhibited regular spherical nanoparticles with high encapsulation efficiency. Pharmacokinetic studies confirmed a prolonged half-life (t_1_/_2_) and delayed mean residence time (MRT), accompanied by a 2.8-fold increase in relative oral bioavailability. Tissue distribution studies revealed sustained drug levels in the liver, while delayed elimination was observed in the kidney. In the CCl4-induced hepatotoxicity study, the activities of hepatic CAT, T-AOC, GSH-Px, SOD, and GSH were elevated, whereas serum biological markers ALT, AKP, and AST were reduced after SA-TPGS-Ls treatment. Histopathological studies established that SA-TPGS-Ls remarkably improved the hepatic tissues status [124]. The protective effect of SA-loaded AgNPs on endoplasmic reticulum stress-mediated apoptosis during ovarian ischemia–reperfusion injury (IR) in rats was evaluated. Treatment with either AgNPs or SA reduced inflammation, apoptosis, and ER stress markers in IR-induced rats. Nevertheless, notable effects were noted in rats upon treatment with SA plus AgNPs. These findings advocate that SA-loaded silver nanoparticles have an auspicious therapeutic application in ovarian ischemia–reperfusion (IR) injury [125]. A study was performed to evaluate the impact of SA nanoparticles (SANPs) on hyperglycemia, mainly in relation to advanced glycation end products, ROS, and inflammation. The administration of SANPs caused a substantial improvement in the activity of glyoxalase-1, glyoxalase-2, and hexokinase-2. There was a noteworthy decrease in MDA levels along with increased GSH, SOD, and catalase activity [126]. The therapeutic role of zinc oxide-conjugated syringic acid (ZnO-SYR) was explored in a mouse model of lung cancer. Treatment by ZnO-SYR notably improved the body weight and lowered serum enzyme levels compared to BAP-treated animals. Additionally, cytokine analysis confirmed a noteworthy reduction in inflammatory mediators, including TNF-α, IL-1β, and IL-6. These biochemical results were supported by histopathological observations, which showed an improved lung tissue architecture in ZnO-SYR-treated mice comparative to those with BAP-induced lung cancer [127]. Qu-SA-loaded chitosan-caseinate nanoparticles were made with a particle yield of 72.4%. The measured loading efficiency and capacity were assessed at 76.1 and 40.3% for quercetin, and 62.4 and 22.7% for SA, respectively. The nanoparticles confirmed noteworthy antioxidant activity, as shown by the IC50 value of 12.5 µg/mL for DPPH radical scavenging potential. These findings indicate that chitosan-caseinate nanoparticles are effective carriers for improving the delivery and bioactivity of SA and quercetin, offering potential applications in functional foods and dietary supplements [128]. Another study aimed to deliver SA with a nanocarrier and improve its function. mPEG-PLGA-PLL (PEAL) nanoparticles were utilized to deliver SA. SA-PEAL showed good storage stability, blood compatibility, and biocompatibility, and slowly released SA. SA-PEAL meaningfully improved the proliferation and migration ability of Schwann cells and the functional recovery of injured sciatic nerves [129]. A study made syringic acid-loaded nanostructured lipid carriers (SA-NLCs) to treat breast cancer. The SA-NLCs showed spherical shapes with a particle size and zeta potential of 142.7 ± 4 nm and −14.5 ± 3.6 mV, respectively. Improved cytotoxicity of the optimized SA-NLCs (IC50: 3.8 ± 0.2 μg/mL) was observed as compared to free SA (IC50: 14.5 ± 1.2 μg/mL) on MCF-7 human breast carcinoma cell lines. Therefore, the improved cytotoxicity of SA-NLCs on MCF-7 cell lines can offer an appropriate approach for breast cancer treatment [130]. SA-loaded TPGS liposome (SA-TPGS-Ls) was prepared to improve the oral bioavailability of SA. The results showed that SA-TPGS-Ls formed uniform, spherical nanoparticles with an encapsulation efficiency of 96.48 ± 0.76%. Pharmacokinetic studies indicated a delayed mean residence time (MRT) and prolonged half-life (t_1_/_2_), while the relative oral bioavailability increased by 2.8-fold. Tissue distribution analysis revealed that SA-TPGS-Ls maintained drug concentrations in the liver, and delayed elimination was also noted in the kidneys. In the CCl4-induced hepatotoxicity study, the activities of hepatic antioxidant enzymes were greatly increased, while serum biological markers ALT, AKP, and AST were reduced after treatment of mice with SA-TPGS-Ls. Histopathological findings established that SA-TPGS-Ls could notably improve the status of hepatic tissues [124].

The study was performed to develop an SA nanoemulsion (SA-NE) through spontaneous emulsification as an anti-psoriatic therapy by the dermal route. Results exhibited that SA-NE comprising a mixture of lauroglycol 90, 10% tween80 (F5), and limonene was selected as the ideal formula, offering a stable nanoemulsion for a 2-month period, displaying a droplet size of 177.6 ± 13.23 nm, zeta potential of −21.23 ± 0.41 mV and polydispersity index of 0.16 ± 0.06. A high SA% in several skin strata and no dermal irritation were observed for limonene-based SA-NE, and it showed high in vitro anti-inflammatory potential. A preclinical study established that limonene-based SA-NE is effective in lessening psoriasis-like skin lesions against imiquimod-induced psoriasis in rats. Clinically promising anti-psoriatic activity was stated as all patients receiving F5 experienced a better clinical improvement as well as response to therapy, attaining ≥50% lessening in PASI scores versus only 35% responders in the Dermovate^®^ cream group [131].

Most available pharmacokinetic and bioavailability studies have been conducted in animals. Further studies are required to establish the absolute oral bioavailability of SA, characterize its plasma and tissue metabolites, and find the major metabolite. Well-designed human pharmacokinetic studies are vital before SA can be considered a clinically viable therapeutic candidate.

Although syringic acid (SA) has confirmed substantial pharmacological action in experimental models in vivo and intro, its development as a conventional drug molecule remains at an initial stage. Substantial efforts have been directed toward the modification of SA and the development of advanced delivery systems to overcome its pharmacokinetic and physicochemical limitations. Some SA derivatives have been investigated as potential therapeutic molecules. Using Surflex-Dock interfaced with SYBYL, the docking affinities of SA and its proposed derivatives to the 20S proteasome were evaluated. Several derivatives were virtually designed, and five derivatives—benzyl 4-hydroxy-3,5-dimethoxybenzoate (1), benzyl 4-(benzyloxy)-3,5-dimethoxybenzoate (2), 3′-methoxybenzyl 3,5-dimethoxy-4-(3′-methoxybenzyloxy)benzoate (3), 3′-methoxybenzyl 4-hydroxy-3,5-dimethoxybenzoate (4), and 3′,5′-dimethoxybenzyl 4-hydroxy-3,5-dimethoxybenzoate (5)—were selected based on their favorable docking scores. These compounds were then synthesized and assessed for their anti-mitogenic activity against human colorectal cancer, breast cancer, malignant melanoma cells, as well as normal human fibroblast cells. It was noted that derivatives **2**, **5**, and **6** exhibited a selective dose-dependent anti-mitogenic role in human malignant melanoma cell lines, with slight cytotoxicity on colorectal and breast cancer cells as well as normal human fibroblast cells. Derivatives **2**, **5** and **6** meaningfully inhibited the numerous proteasomal chymotrypsin, PGPH, as well as trypsin-like activities. The derivatives also arrested the growth of HTB68 cells at the S and G2 phases, correspondingly. Also, derivatives **2**, **5**, and **6** suggestively promoted apoptosis in both HTB66 and HTB68 cells [132]. The study was designed to develop xanthine oxidase inhibitors along with the antioxidant role. It designed and synthesized syringic acid derivatives hybridized with alcohol and amines to make ester and amide linkage through molecular docking. The synthesized compounds were assessed for their xanthine oxidase inhibitory and antioxidant potential. The study confirmed that SY3 shows good xanthine oxidase inhibitory action. All the compounds exhibited good antioxidant activity. The enzyme kinetic studies on SA derivatives exhibited an inhibitory effect on XO capability in a competitive manner, and SY3 was revealed as the furthermost active derivative. Molecular simulation showed that new SA derivatives interacted with the amino acid residues SER1080, GLN1194, PHE798, ALA1078, ARG912, GLN 767, and MET1038 located inside the binding site of XO [133].

## 7. Safety and Toxicity of Syringic Acid

Safety and toxicity evaluation of any drug or bioactive compound is an important step before human administration. Wide-ranging toxicological assessment helps identify adverse reactions, target-organ toxicity, and safe dosage ranges. Syringic acid has generally been reported to be safe based on previous studies. Toxicological evaluations have not shown significant adverse effects at tested doses. In a subacute toxicity study, both treatment and satellite groups received SA at a dose of 1000 mg/kg/day orally for 14 days. The toxicological effects of SA were assessed by checking clinical signs of toxicity, mortality, and changes in body weight. The results exhibited that SA caused no substantial adverse effects on body weight, food intake, erythropoiesis, or leukopoiesis. Additionally, no noteworthy abnormalities were observed in internal organs based on biochemical and hematological parameters, relative organ weights, or histopathological investigations. These findings indicate that SA has a promising safety profile when administered for a limited period [134]. However, no clinical studies in humans are available to confirm its safety and toxicity. The available evidence is limited to preclinical studies. Therefore, further human studies are vital to establish its clinical relevance.

## 8. Conclusions, Limitations and Future Prospectives

Polyphenols, including phenolic compounds, are widely present in fruits and vegetables and are well recognized for their biological activities. Epidemiological evidence shows that consuming diets rich in antioxidants as well as anti-inflammatory compounds may markedly reduce the risk of disease development and progression. In this regard, syringic acid has emerged as an important naturally occurring phenolic acid due to its promising therapeutic potential in disease prevention and management. Numerous experimental studies have demonstrated the hepatoprotective, cardioprotective, nephroprotective, and neuroprotective effects of syringic acid through the regulation of oxidative stress, suppression of inflammatory mediators, and maintenance of cellular integrity. In addition, preclinical investigations have confirmed its beneficial role in respiratory, digestive, metabolic, and reproductive system-associated complications through modulation of multiple biochemical and molecular pathways. Syringic acid has also been shown to play a role in eye and joint health, further supporting its pharmacological significance. Moreover, its versatile role in cancer prevention and therapy has been reported through the regulation of cellular proliferation, apoptosis, angiogenesis, and other signaling pathways associated with tumor development.

Despite its noteworthy therapeutic potential demonstrated in preclinical models, the clinical application of syringic acid remains limited due to its poor bioavailability, rapid metabolism, and limited pharmacokinetic characterization. Furthermore, the current literature lacks full information on the optimal dosage, long-term safety, and precise molecular mechanisms. Most available evidence comes from preclinical studies, while well-designed human clinical trials remain scarce. Future research should be conducted on the bioavailability of syringic acid using advanced drug delivery systems to enhance its stability, absorption, and tissue-targeting efficiency. Wide-ranging in vivo investigations and clinical studies are vital to confirm its therapeutic efficacy, safety in humans, and potential mechanism of action. Moreover, further studies should explore its synergistic interactions with other compounds/drugs.

## Figures and Tables

**Table 5:** Hepatoprotective effects of syringic by reducing serum liver enzyme levels, attenuating oxidative stress and lipid peroxidation, suppressing inflammatory cytokines, and improving histopathological architecture of liver tissue.

	Study Model	Doses	Findings	Refs.
Hepatoprotective effects	Ethanol-induced hepatotoxicity in mice	40 or 50 mg/kg	° SA lowered hepatic inflammatory infiltration	[76]
	Acetaminophen-induced hepatotoxicity in rats	25, 50 and 100 mg/kg	° SA exhibits hepatoprotective and antihyperlipidemic activity	[77]
	Sodium valproate-induced liver injury in rats	40 and 80 mg/kg	° Liver transaminase activities and oxidative stress inhibited by SA ° Pro-inflammatory markers decreased by SA	[78]
	Sodium arsenite-induced hepatotoxicity in mice	10 and 25 mg/kg	° SA reduced levels of liver enzymes and raised levels of antioxidant enzymes and caspase-3 expression	[79]
	CCl_4_-induced liver injury in mice	10 mg/kg	° SA suppressed hepatic fibrosis	[80]
	Acetaminophen-induced hepatotoxicity in Wistar rats	50 mg/kg	° SA reversed APAP-induced hepatotoxicity in rats	[81]
	DOX-induced hepatic injury in rats	25, 50, or 75 mg/kg	° SA therapy reduced hepatotoxicity by reducing oxidative stress and inflammation	[9]
	DMN-induced hepatic injury in rats	50 mg/kg	° SA treatment diminished liver enzymes and inflammatory marker levels	

Abbreviations: SA, syringic acid; CCl_4_, carbon tetrachloride; DOX, doxorubicin; DMN, dimethylnitrosamine.

**Table 8:** The antimicrobial activities of syringic acid (SA) against various microorganisms and pathogens. Syringic acid revealed biological activity, including antibacterial, antifungal, and antibiofilm properties. The antimicrobial mechanisms stated include disruption of cell membrane integrity and inhibition of biofilm formation.

	Activity	Organism/Pathogen	Findings	Refs.
Syringic acid	Antibacterial	*Cronobacter sakazakii*	° Bacterial growth retarded by SA, and cell membrane dysfunction caused by SA ° Intracellular ATP concentration decreased, cell membrane hyperpolarization and changes in cellular morphology	[113]
	Antifungal	*G. boninense*	° Strong fungitoxic activity observed at 0.5 mg/mL	[114]
	Antibacterial	*Escherichia coli* O157:H7, *Salmonella enterica* serovar Typhimurium, *Listeria monocytogenes*, and *Staphylococcus aureus*	° Syringic acid (SA) and mild heat (MH) inactivated diverse foodborne pathogens through multi-targeted cellular damage	[115]
	Antibiofilm	Methicillin-resistant *Staphylococcus epidermidis*	° Biofilm formation reduced up to 80% and EPS production up to 55% with SA	[116]

**Table 9:** Synergistic activities of syringic acid in combination with bioactive compounds and therapeutic drugs. Co-treatment with SA and other drugs/compounds demonstrated an effective synergistic agent that enhances the efficacy of therapeutic compounds through modulation of oxidative stress, inflammation, and cellular pathways.

	Compounds/Drugs	Study Types	Activity	Outcomes	Refs.
Syringic acid	Doxorubicin	In vitro	Role in endometrial cancer	° Co-treatment with SA as well as doxorubicin established an additive inhibitory potential ° Co-treatment suppressed cell migration	[94]
	Zinc sulfate	In vivo	Antidiabetic and antioxidative effects	° Complexing zinc (II) and SA improved their anti-hyperglycemic action ° The complex reduced systemic and tissue lipid peroxidation	[118]
	Gigantol		Prevention of diabetic cataract	° Combined administration enhanced anti-cataract efficacy	[119]
	Resveratrol	In vivo	Cardioprotection	° COMB suppressed MI and exhibited cardioprotection	[61]
		In vivo cardioprotection	Cardioprotection	° COMB pretreatment caused cardioprotection and ameliorated cardiotoxicity	[61]

Abbreviations: SA, syringic acid; Dox, doxorubicin; MI, myocardial infarction; COMB, combination treatment (as reported in study).

## Data Availability

No new data were created or analyzed in this study. Data sharing is not applicable to this article.
